# Octopus-Inspired Self-Adaptive Hydrogel Gripper Capable of Manipulating Ultra-Soft Objects

**DOI:** 10.1007/s40820-025-01880-4

**Published:** 2025-08-19

**Authors:** Yixian Wang, Desheng Liu, Danli Hu, Chao Wang, Zonggang Li, Jiayu Wu, Pan Jiang, Xingxing Yang, Changcheng Bai, Zhongying Ji, Xin Jia, Xiaolong Wang

**Affiliations:** 1 State Key Laboratory of Solid Lubrication, Lanzhou Institute of Chemical Physics, Chinese Academy of Sciences, Lanzhou, 730000 People’s Republic of China; 2 School of Chemistry and Chemical Engineering/State Key Laboratory Incubation Base for Green Processing of Chemical Engineering, Shihezi University, Shihezi, 832003 People’s Republic of China; 3 School of Mechanical Engineering, Lanzhou Jiaotong University, Lanzhou, 730070 People’s Republic of China

**Keywords:** Octopus sucker structure, Self-adaptive gripper, Supramolecular hydrogel, Underwater switchable attachment, Nondestructive manipulating

## Abstract

Octopuses, due to their flexible arms, marvelous adaptability, and powerful suckers, are able to effortlessly grasp and disengage various objects in the marine surrounding without causing devastation. However, manipulating delicate objects such as soft and fragile foods underwater require gentle contact and stable adhesion, which poses a serious challenge to now available soft grippers. Inspired by the sucker infundibulum structure and flexible tentacles of octopus, herein we developed a hydraulically actuated hydrogel soft gripper with adaptive maneuverability by coupling multiple hydrogen bond-mediated supramolecular hydrogels and vat polymerization three-dimensional printing, in which hydrogel bionic sucker is composed of a tunable curvature membrane, a negative pressure cavity, and a pneumatic chamber. The design of the sucker structure with the alterable curvature membrane is conducive to realize the reliable and gentle switchable adhesion of the hydrogel soft gripper. As a proof-of-concept, the adaptive hydrogel soft gripper is capable of implement diversified underwater tasks, including gingerly grasping fragile foods like egg yolks and tofu, as well as underwater robots and vehicles that station-keeping and crawling based on switchable adhesion. This study therefore provides a transformative strategy for the design of novel soft grippers that will render promising utilities for underwater exploration soft robotics.

**Supplementary Information:**

The online version contains supplementary material available at 10.1007/s40820-025-01880-4.

## Highlights

- 3D printable supramolecular hydrogels with tunable mechanical properties and stiffness adaptability were enabled by strong and weak H-bonding cooperative interactions and microphase separation.
- Sucker structure with an alterable membrane was designed and fabricated with 3D printing  to realize reliable and gentle switchable adhesion.
- Octopus-inspired hydrogel gripper that is capable of delicately handling ultra-soft underwater objects in the form of nondestructive surface release was achieved.

**Supplementary Information:**

The online version contains supplementary material available at 10.1007/s40820-025-01880-4.

## Graphical abstract

**Figure:**
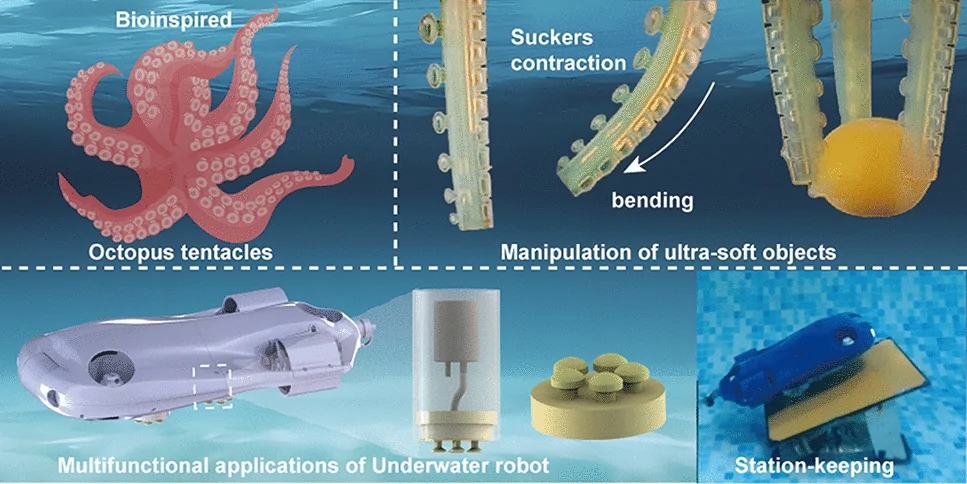

## Introduction

Cephalopods such as octopuses in the ocean exhibit exceptional underwater adaptive maneuvering capabilities through dynamic sucker-actuated arm systems. This biomechanical architecture is conducive to switchable adhesion and dexterous object manipulating on a variety of substrates with diverse geometries, while maintaining nondestructive contact invasiveness. To this end, this unique biomechanical synergy has inspired considerable advances in aquatic gripper technologies, spanning switchable underwater adhesion [1–5], suction-based end-effectors [6, 7], and soft robotic manipulators [8, 9]. Compared to their biological prototypes, however, up-to-the-minute artificial gripper is still constrained by fundamental determinants such as environmental adaptability and operational polyvalence [10, 11]. Excitingly, the up-to-date integration of the bionic suction cup structures and soft arms has facilitated the development of octopus-inspired grippers that achieve more diverse functions in intricate underwater operations, including climbing [12, 13], swimming [14, 15], and adaptive grasping of various targets [16–18]. Nonetheless, it can be exceedingly challenging to grasp many soft and fragile objects such as tofu and egg yolks, as well as apparatuses with irregular shapes and variable curvatures, especially for underwater scenario applications. Based on this, it is critical to develop an adhesion gripper that can adapt to specific shape requirements, achieve robust gripping capabilities, and possess an active release mechanism to prevent damage, while also presenting significant challenges for material design and structural fabrication.

The actuation mechanisms and structural design, as everyone knows, establish the functional foundations of mechanically adhered soft grippers, because their ultimate dexterous maneuverability and service life depend predominantly on the mechanical properties of materials and their adaptability to the circumstances [11, 18, 19]. Up to this point, soft grippers are substantially fabricated from stimulus-responsive smart materials, which showcase fast actuation responsiveness and multimodal deformation capabilities [20, 21]. Nevertheless, such soft actuation grippers have long encountered perpetual challenges in achieving adaptive gripping and steerable releasing, especially in underwater scenarios. In contrast, soft grippers powered by pneumatic or hydraulic pressure and with bionic structures are well known for their robust gripping performance and adaptability in complex manipulation [15, 18, 22, 23]. However, these soft grippers are primarily made of hydrophobic silicon-based materials, which are restricted by insufficient driving force and circumscribed deformation range due to factors such as osmotic pressure in underwater or deep-sea environments. Accordingly, achieving a paradigm shifts from traditional hydrophobic silicone-based systems to hydrophilic soft matters is essential for the manufacture and application of soft grippers for controllable manipulation of underwater objects.

Unlike hydrophobic elastomers with intrinsic entropy elasticity, the flexibility of hydrogels with wet, adhesive, and lubricious characteristics relies on the presence of moving water molecules within the crosslinked polymer networks [24–28]. Consequently, such dependency of hydrogels has attracted great interest in the fields of biolubrication [29–31], underwater adhesion [25, 32–34], and soft actuators [33–37], particularly in pneumatic/hydraulic actuation systems that are compatible with aquatic environments [38, 39]. How to replicate biological structure and mechanical adaptability is crucial to the invention of bionic and high-performance hydrogel grippers for the manipulation of underwater objects. As luck would have it, vat photopolymerization three-dimensional (3D) printing, as an emerging fabrication technology, offers a huge revolution in the construction of hydrogel devices with intricate and ultra-precise biomimetic architectures through a layer-by-layer approach [39–44]. For instance, Mishra et al. [45] developed a finger-like fluidic hydrogel actuator based on this 3D printing, which can open and close autonomically in response to thermal fluctuation. In their work, nevertheless, the unsatisfactory mechanical properties and swelling stability of thermos-responsive hydrogel material constrain their widespread application in underwater scenarios. Furthermore, Wang et al. [16] reported a bionic octopus sucker gripper constructed by harnessing dual-network hydrogel and digital light processing (DLP) 3D printing, which is able to flexibly adhere to various underwater objects based on negative pressure and water film sealing. Notwithstanding, the stiffness and toughness of this hydrogel are too high for the gripper to achieve a satisfactory curvature bend under hydraulic actuation. Meanwhile, the proposed hydrogel suction cup structure fails to achieve switchable adhesion performance; thereupon, it is difficult to integrate adhesion and deadhesion functions. Thus, an approach to achieve both intricate bionic adhesion structures and tunable mechanical properties is urgent for contriving bionic structural grippers with switchable adhesion based on hydraulic actuation.

Inspired by the infundibulum microstructure of an octopus sucker, herein a new tactic of engineering self-adaptive hydrogel gripper (SAHG) with switchable adhesion that is capable of manipulating ultra-soft objects is proposed. To achieve SAHG with a combination of the aforementioned features, a supramolecular hydrogel with tunable mechanical properties and stiffness adaptability enabled by the strong and weak H-bonding cooperative interactions and the microphase separation. Then, vat photopolymerization 3D printing was utilized to construct high-precision hydrogel bionic sucker that is composed of a tunable curvature membrane, a negative pressure cavity, and a pneumatic chamber, in which the hydrogel curvature membrane regulated the deadhesion of the suction cups. The proposed suckers with curvature membranes showcase switchable adhesion to various rough surfaces such as silicon, glass, S304, and nylon. To further underscore the nondestructive adhesion practicability, a hydraulically actuated hydrogel gripper equipped with bionic suckers was manufactured to adaptively manipulate various underwater objects, including numerous substances with complex curved surfaces, and even extremely fragile foodstuffs such as egg yolks and tofu blocks. We believe that the proposed adaptive adhesion structure design strategy holds significant application potential in the field of hydrogel-based smart soft grippers as well as climbable and moveable robots.

## Experimental Section

### Materials

Acrylamide (AAm, 98%, Kermel, Tianjin, China), acrylic acid (AAc, > 99%, TCI, Tokyo, Japan), acryloyl chloride (98%, Macklin, Shanghai, China), semicarbazide hydrochloride (98%, Energy Chemical, China), diethyl ether (99.5%, Kelong Company, China), potassium carbonate anhydrous (K_2_CO_3_, 99%, Aladdin, Shanghai, China), lithium phenyl-2,4,6-trimethylbenzoylphosphinate (LAP, 95%, Sigma-Aldrich), tartrazine (95%, Aladdin, Shanghai, China), dimethyl sulfoxide (DMSO, 98%, Kermel, Tianjin, China). Deionized (DI) water was prepared in the laboratory. All chemical reagents were used without further purification.

### Synthesis of N-Acryloylsemicarbazide (NASC)

Semicarbazide hydrochloride (31.75 g) with 30 mL deionized water (DI), 168 mL of cool K_2_CO_3_ solution (2 M), and 90 mL cold diethyl ether were successively added into a 500 mL round-bottom flask. Then, 28.50 g of acryloyl chloride in 120 mL diethyl ether was added drop wise to the above solution under stirring at 0 °C for ≈4 h. After that, the white precipitate was collected by filtration and washed in cold water to get the crude product. The crude product was dissolved in DI and stirred for 1 h at 95 °C, subsequently, the undissolved precipitate was removed by centrifuge, and the supernatant was freeze-dried to obtain the final NASC monomer.

### Preparation of Hydrogel

The hydrogels with different compositions ratios were fabricated by the photo-initiated radical polymerization under the irradiation of 405 nm UV light at room temperature. A certain mass of the monomers NASC, AAm, and AAc were dissolved in the mixed solvent of DMSO and DI water (Table S1); then photoinitiator (LAP, 0.5 wt% of the total monomers) and tartrazine (0.3 g L^−1^) were added into beaker and mixed by magnetic stirring for 60 min at nitrogen atmosphere. The hydrogel precursor solution of 15 mL was transferred into a reaction cell consisting of a transparent acrylic plate (10 cm × 10 cm × 1 cm) and was triggered by irradiation under 405 nm UV light to form the as-prepared hydrogels. The as-prepared hydrogels were thoroughly immersed with DI water for 10 days to completely remove DMSO.

### 3D Printing the Hydrogel Suckers and Robotic Gripper

A photocurable hydrogel ink solution was prepared by dissolving certain amounts of NASC, AAm, AAc, and LAP in the mixed solvent of DMSO and DI water (Table S1), and tartrazine was added as photoabsorber at a concentration of 0.3 g L^−1^. Afterward, hydrogel ink solution was loaded into the resin tank and mounted onto a commercial stereolithography 3D printer (SLASH, UNIZ, China). The printing system operates with a 405 nm UV light source with a light intensity of 300 mW cm^−2^. Different 3D sucker or gripper models in stereolithography (STL) files were transferred to a stereolithography 3D printer (SLASH, UNIZ, China) and were sliced in the Z-direction before printing. The 3D printing parameters were set as follows: The thickness of the single layer is 100 μm, the curing time is 15 ~ 25 s, and the platform temperature is room temperature for all experiments. After printing, the phantoms were immersed in deionized water for 10 days to obtain a hydrogel phantom.

### Mechanical Properties Testing

The tensile tests were performed on a universal material testing machine (EZ-Test, SHIMADZU, Japan) with a 500 N load cell. The rectangular gels and hydrogels were used for the tests. The thickness and width of the specimen were 1.0 ~ 1.3 mm. The samples were fixed between the double-sided tapes to fasten the grips. The gauge length between the clamps was 25 mm. Samples were stretched at a rate of 100 mm min^−1^ until breaking. The tensile breaking stress and breaking strain were calculated from the slope of the stress–strain curve. The Young's modulus (E, MPa) was calculated from the slope of the stress–strain curve, ranging from 5 to 15% of the strain. Work of fracture (toughness), W (MJ m^−3^), was defined as the area covered by the stress–strain curves as follows:

1 $$W=\int\limits_{\varepsilon =0}^{\varepsilon ={\varepsilon }_{\mathrm{b}}}{\sigma }_{\mathrm{b}}\mathrm{d}\varepsilon$$

where ${\sigma }_{\mathrm{b}}$ and ${\varepsilon }_{\mathrm{b}}$ were the corresponding stress and strain at breaking, respectively. The measurement of each sample was repeated at least five times.

To examine the fatigue resistance of the hydrogels, one hundred consecutive cyclic loading–unloading tests with different strain were performed. At a strain rate of 100 mm min^−1^, a rectangular specimen (length 40 mm × width 8 mm × thickness 1 mm) was stretched, released, and then stretched. To prevent water evaporation, the hydrogels were submerged in a water bath during the measurements. At least three separate tests were measured for each sample. The meaning was calculated, and the standard deviation was obtained as error bars.

The sample with a prescribed width *w* = 10 mm and length *w* = 40 mm was prepared. An initial notch of 20 mm was made in the middle of the sample along the length direction with a cutter. During testing, one leg of the sample was clamped to the base, and the other was clamped to the crosshead, which was displaced at a velocity of 100 mm min^−1^ in the water. After testing, the tearing strength–displacement curves were obtained to calculate the tearing energy of samples by the following equation:

2 $${E}_{\mathrm{s}}=\frac{{\int }_{0}^{L}F\mathrm{d}l}{t{L}_{\mathrm{bulk}}}$$

where *F* was the tearing force, *t* was the sample thickness, *L* was the displacement, and *L*_bulk_ was the projected crack length.

### Rheology Behavior

The Haake Mars 60 rheometer was also utilized to study the UV-curing reaction kinetics of the inks. Its UV light source was 405 nm, 3 W, and the distance of the light source from the hydrogel was 1.5 cm. The ink was exposed to UV light for 200 s, and the *G*' and *G*'' were measured.

### Adhesion Tests

Underwater adhesion strength of the octopi-inspired hydrogel suckers was measured by using a universal material testing machine (EZ-Test, SHIMADZU, Japan) with a 500 N load cell. All tests used custom fixtures. The crosshead speed was set to be 10 mm min^−1^ in the tensile mode to obtain the load force–displacement curve. To investigate the effects of preload on the adhesion strength, the applying load was set to 0.2, 0.5, 1, 2, and 3 N with a contact time of 60 s. To investigate the effect of negative pressure on adhesion strength, the applied negative pressure was set at − 11.5, − 20, − 26.8, − 32, and − 36 kPa. To investigate the nonspecific adhesion of the hydrogel suckers, different substrates and surface roughnesses were used. For the adhesion test, the applied load was set as 1 N, negative pressure was set as − 36 kPa, and the contact time between two surfaces was 60 s.

### Hydraulic Actuation of Hydrogel Suckers and Robotic Gripper

The hydraulic hydrogel actuator is powered by pressurized water supplied from multiple customized programmable high-throughput syringe pumps. The pressurized water flows into the hydrogel actuator through hydraulic connections, including silicone tubing and metal needles. The actuator is inflated and deflated through the injection and extraction of water via the syringe pump, which is programmed using custom code. The speed of the actuator is regulated by adjusting the flow rate of the water supply in the syringe pump. Synchronized actuation of the membrane and arm of the hydrogel gripper is accomplished using two sets of independently controlled syringe pumps. Air bubbles within the hydraulic chamber of the hydrogel actuator can be effectively eliminated by squeezing or degassing the actuator while submerged under water.

### Pressure–Volume Curves

To analyze mass transport characteristics of the hydraulic hydrogel actuators, pressure–volume hysteresis curves were generated by hydraulic inflation and deflation of the actuators in water. The syringe pump was connected to the hydrogel actuators that were clamped and submerged in water in a vertical position, and the pressure sensor was also connected via T-junction. Water was infused into the hydrogel actuator with sufficiently low supply flow rate until the amount of supplied water reached the actuation volume; then the actuator was deflated by withdrawing water from it. Once the actuator had completed one full cycle and the pressure returned to zero, the same cycle was repeated at least three times to assure reproducibility. The recorded pressure was plotted against volume to form pressure–volume hysteresis curves, and energy analysis was performed using the area under the pressure–volume hysteresis curves.

## Results and Discussion

### Design and Fabrication of Octopus-Inspired Adaptive Hydrogel Soft Gripper

**Fig. 1:**
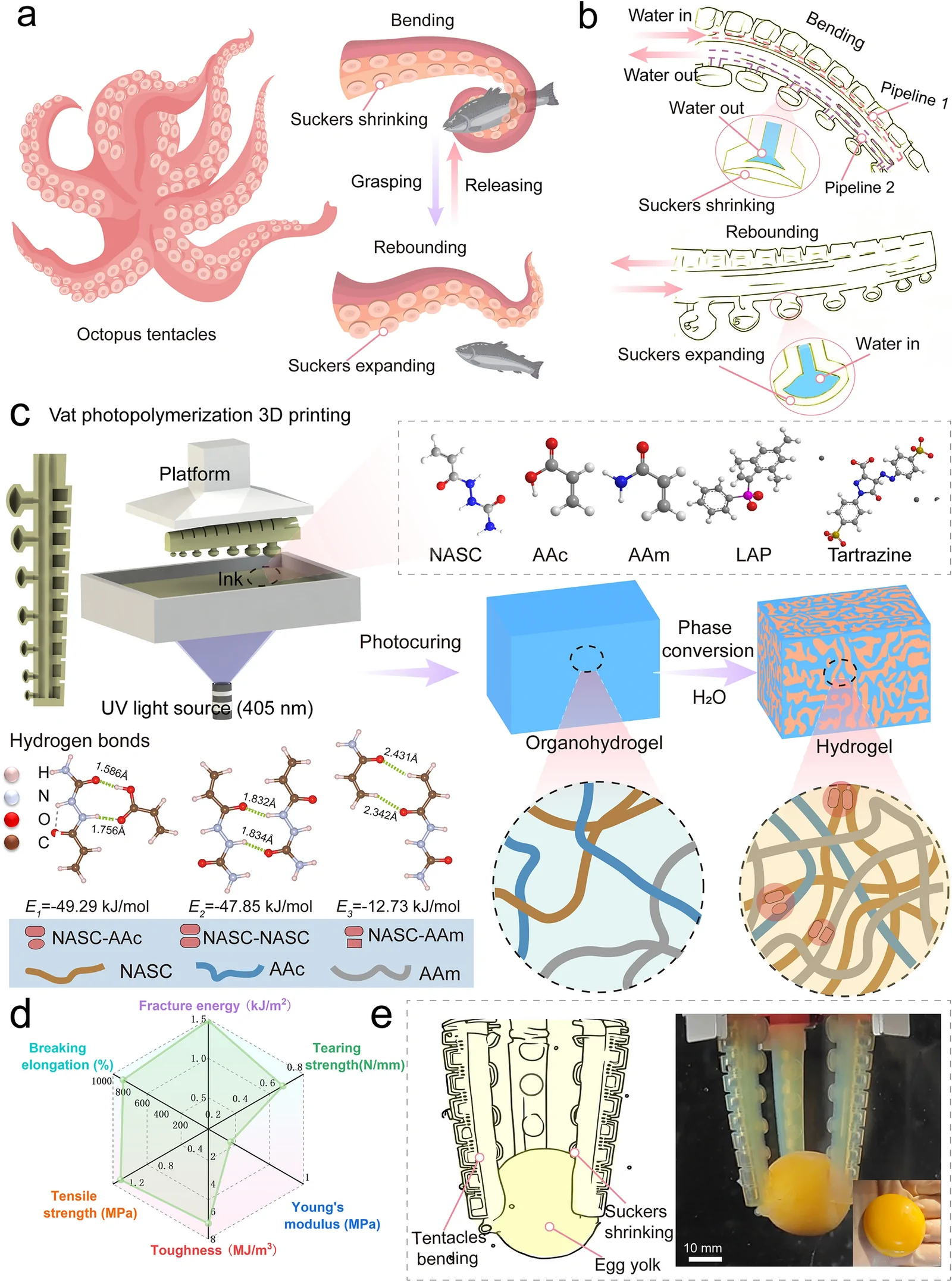
Design and fabrication of octopus-inspired self-adaptive hydrogel soft grippers. **a** Schematic diagram of an octopus's predatory behavior with suckers and tentacles. **b** Schematic illustration of the design principle and hydraulic drive mechanism of the octopus-inspired hydrogel soft gripper. **c** Photosensitive ink composition, reaction mechanism, and multiple hydrogen bonding interactions of supramolecular hydrogels, as well as the fabrication process of the octopus-inspired hydrogel soft grippers. **d** The optimized mechanical properties of supramolecular hydrogel for adaptive soft gripper fabrication, spanning strength, ductility, Young's modulus, fracture energy, and toughness. **e** Schematic diagram and photograph of an octopus-inspired hydrogel gripper handling an underwater ultra-soft object like an egg yolk

Inspired by the unique characteristics of the octopus's soft tentacles and unique adaptive sucker structure, which mainly functions as adhesion and desorption, we developed a hydrogel soft gripper with self-adaptive manipulation functionality by coupling vat photopolymerization 3D printing and mechanically adaptable supramolecular hydrogels (Fig. 1a, b). It should be noted that the work of the suction cups (contraction and expansion) and tentacles (bending and rebounding) of the hydrogel soft gripper are controlled by two separate pipeline pathways. To formulate such a proof-of-concept supramolecular hydrogel photocurable ink, as schematized in Fig. 1c, multiple components including N-acryloylsemicarbazide (NASC), acrylic acid (AAc), acrylamide (AAm) as monomers, LAP as water-soluble photoinitiator, and water-soluble dye tartrazine as photoabsorber are dissolved into a mixed solvent of dimethyl sulfoxide(DMSO) and H_2_O. Furthermore, density functional theory (DFT) validated that the binding energy of NASC to AAc, NASC to NASC, and NASC to AAm was − 49.29, − 47.85, and − 12.73 kJ mol^−1^, respectively, underscoring that there is a robust supramolecular network of hydrogen bonding in the hydrogel system to modulate the mechanical properties. Afterward, the photopolymerization kinetics behavior of photosensitive hydrogel ink was further validated by harnessing a rheometer (Fig. S4). It was found that the viscosity and modulus (i.e., storage modulus and loss modulus) of photosensitive hydrogel inks originally increased rapidly with the extension of UV irradiation (405 nm light source) time, and then stabilized at higher levels, indicating that their curing depth was UV irradiation-dependent. Meanwhile, a double bond conversion rate of more than 90% after 16 s of UV irradiation, accentuating that the hydrogel photosensitive ink exhibited superlative photopolymerization performance (Fig. S5). Subsequently, the UV-cured organohydrogel was immersed in DI water for microphase separation and hydrogen bonding reconfiguration to produce supramolecular hydrogels with superior mechanical properties [26]. More importantly, the mechanical properties of supramolecular hydrogels are able to controllably optimize by modulating the interaction of multiple strong and weak hydrogen bonding of monomers, resulting in a soft and tough supramolecular hydrogel suitable for the manufacture of soft grippers. As revealed in Fig. 1d, the optimized supramolecular hydrogel for adaptive soft gripper manufacturing showcases a tensile strength of 1.30 ± 0.07 MPa, a break elongation of 893.88 ± 55.55%, a Young’s modulus of 0.24 ± 0.02 MPa, a fracture energy of 0.65 ± 0.33 kJ m^−2^, and a toughness of 6.87 ± 0.66 MJ m^−3^. It is a wonder that the handling of miscellaneous underwater complex objects is feasible thanks to the cooperative effect of the bionic suction cup structure and the soft tentacles features, especially the gripping and nondestructive releasing of ultra-soft surfaces like egg yolk (Fig. 1e).

### Mechanical Properties Modulation and Structural Evolution of Supramolecular Hydrogels

**Fig. 2:**
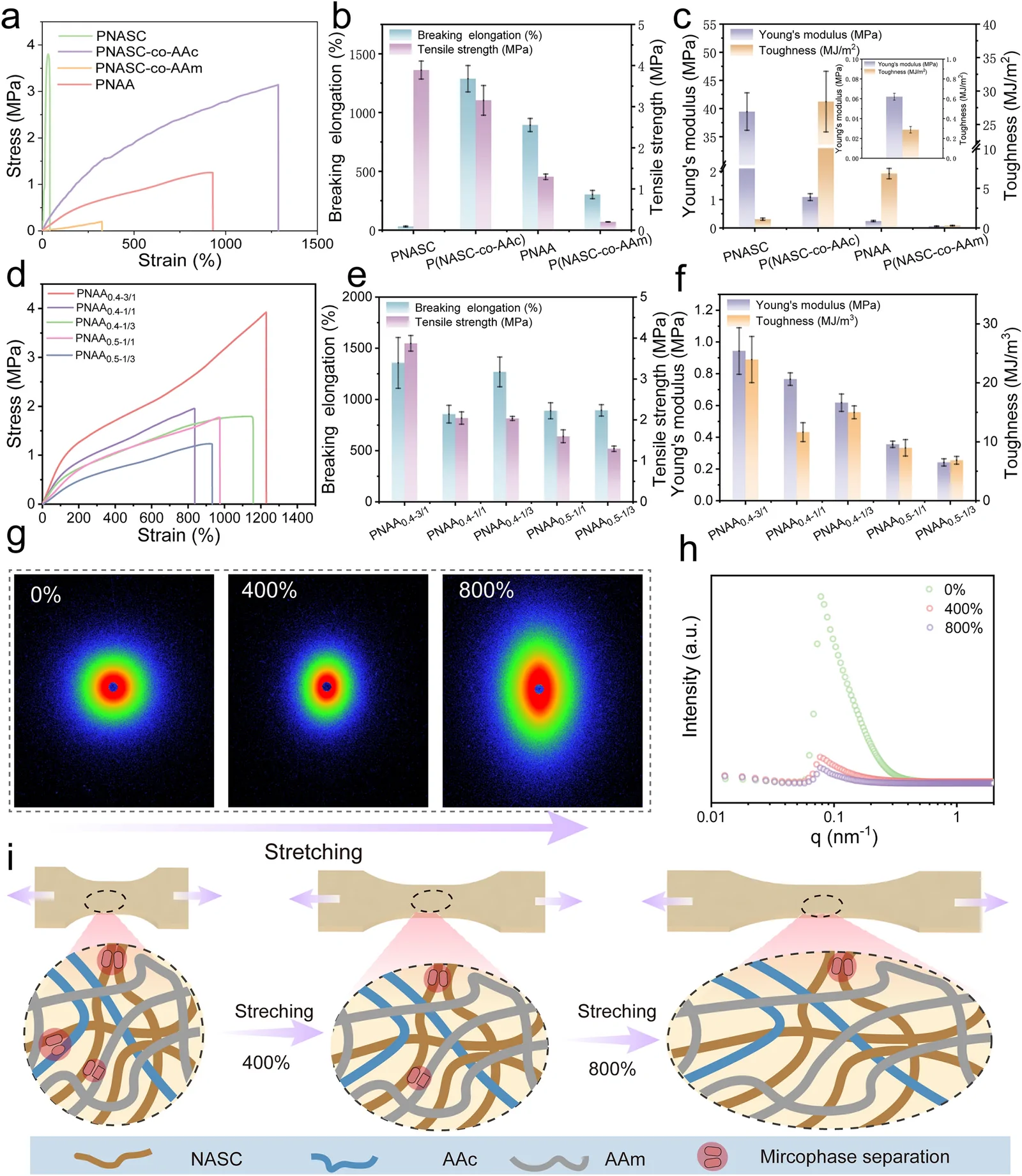
Mechanical properties and structural evolution of supramolecule hydrogels. **a** Representative tensile stress–strain curves, **b** breaking elongation and tensile strength, as well as **c** Young’s modulus and toughness of PNASC, P(NASC-co-AAc), P(NASC-co-AAm), and PNAA hydrogels. **d** Typical tensile stress–strain curves, **e** breaking elongation and tensile strength, as well as **f** Young’s modulus and toughness of PNAA supramolecule hydrogels with different AAm and AAc content and mass ratios. **g** 2D SAXS scattering patterns and corresponding **h** 1D SAXS profiles of a PNAA_0.5–1/3_ hydrogel under different stretching strains. **i** Schematic illustration of the strengthening and toughing mechanism of PNAA hydrogels through microphase separation domains. Error bars represent ± SD (*n* = 3)

Among the various soft actuators, a crucial challenge is to have the suitable mechanical properties to maintain robustness and functionality under cyclic pneumatic/hydraulic actuations without leaking or failure from the bodies and interfaces [41]. To this end, a supramolecular hydrogel with tunable mechanical properties was engineered for adaptive soft gripper fabrication through the cooperative interaction of strong (e.g., between NASC and NASC as well as NASC and AAc with each other) and weak hydrogen bonds (e.g., between NASC and AAm). As shown in Fig. 2a–c, the PNASC hydrogels demonstrated plastic mechanical properties due to the strong H-bonding interactions between amide and urea [46]. Surprisingly, the introduction of AAc is able to effectively modulate the mechanical properties of PNASC hydrogels from plasticity to elasticity, which is due to the presence of noncooperative H-bonding interactions of urea and urea groups as well as urea and carboxyl groups in the P(NASC-co-AAc) hydrogels. Nevertheless, the modulus and toughness of P(NASC-co-AAc) hydrogels are too high to make it unsuitable for the manufacture of soft grippers. To achieve an ultra-soft supramolecular hydrogel, AAm was introduced into NASC to fabricate P(NASC-co-AAm) hydrogels by exploiting the weak H-bonding interactions between the urea and the amino groups. However, the strength, modulus, and toughness of P(NASC-co-AAm) hydrogels deteriorated remarkably due to the excessive presence of weak hydrogen bonds. Thereupon, AAc and AAm with strong and weak H-bonding were simultaneously introduced into NASC, and a series of poly(NASC-co-AAc-co-AAm) hydrogels with tunable mechanical properties by altering density of the H-bonding supramolecular network. For convenience, the resultant supramolecular hydrogels are coded as PNAAx-y/z, where x represents the ratio of the total mass of AAm and AAc to the mass of NASC, and y/z denotes the mass ratio of AAc to AAm. As shown in Fig. 2d–f, the modulus of the PNAA hydrogels decreases dramatically with the increase of the overall AAc and AAm content, while the reduction in tensile strength is not significant. For instance, the Young’s modulus of PNAA_0.5–1/1_ hydrogel decreases from 0.94 ± 0.15 to 0.36 ± 0.002 MPa compared to PNAA_0.4–3/1_, while the tensile strength decreases from 3.87 ± 0.19 to 1.60 ± 0.16 MPa. Additionally, with the increase of the AAc/AAm mass ratio, the Young’s modulus of PNAA hydrogels decreased, which was due to the increase of weak H-bonding interactions between amide and urea groups. To be specific, compared with PNAA_0.5–1/1_ hydrogel, the Young’s modulus of the PNAA_0.5–1/3_ hydrogel decreased from 0.36 ± 0.02 to 0.25 ± 0.02 MPa, while the tensile strength declined from 1.60 ± 0.16 to 1.30 ± 0.07 MPa. Subsequently, small angle X-ray scattering (SAXS) tests were performed to further validate the mechanism of mechanical behavior regulation of supramolecular polymer hydrogels. As shown in Fig. 2g, h, it can be found that the hydrogel has prominent intrinsic microphase separation structure under unstretched states, which is due to the presence of strong and weak multiple H-bonding interactions. When the tensile strain gradually increases to 400%, it can be found that the 2D SAXS scattering pattern is fusiform, and the peak intensity decreases rapidly. This trend is more obvious when the hydrogel is further stretched to 800% strain. These results corroborate the gradual deformation orientation and disintegration of the microphase separation domains of the supramolecular hydrogel along the stretching direction, accompanied by the breaking of some weaker H-bonding network (Fig. 2i). Besides, the hydrogels exhibited superlative tear resistance and crack insensitivity, with rupture energies ranging from 1.48 ± 0.33 to 39.31 ± 3.53 kJ m^−2^ (Fig. S6). The tensile fatigue measurements based on different strains also confirmed that the PNAA supramolecular hydrogel had exceptional fatigue resistance performance (Fig. S7). To sum up, the strong and weak H-bonding interactions of supramolecular hydrogels can be effectively modulated by altering the content of monomers; thereupon, the supramolecular hydrogels have the optimum mechanical properties, including strength, modulus, toughness, and ductility.

### Switchable Adhesion of Octopus-Inspired Hydrogel Sucker

**Fig. 3:**
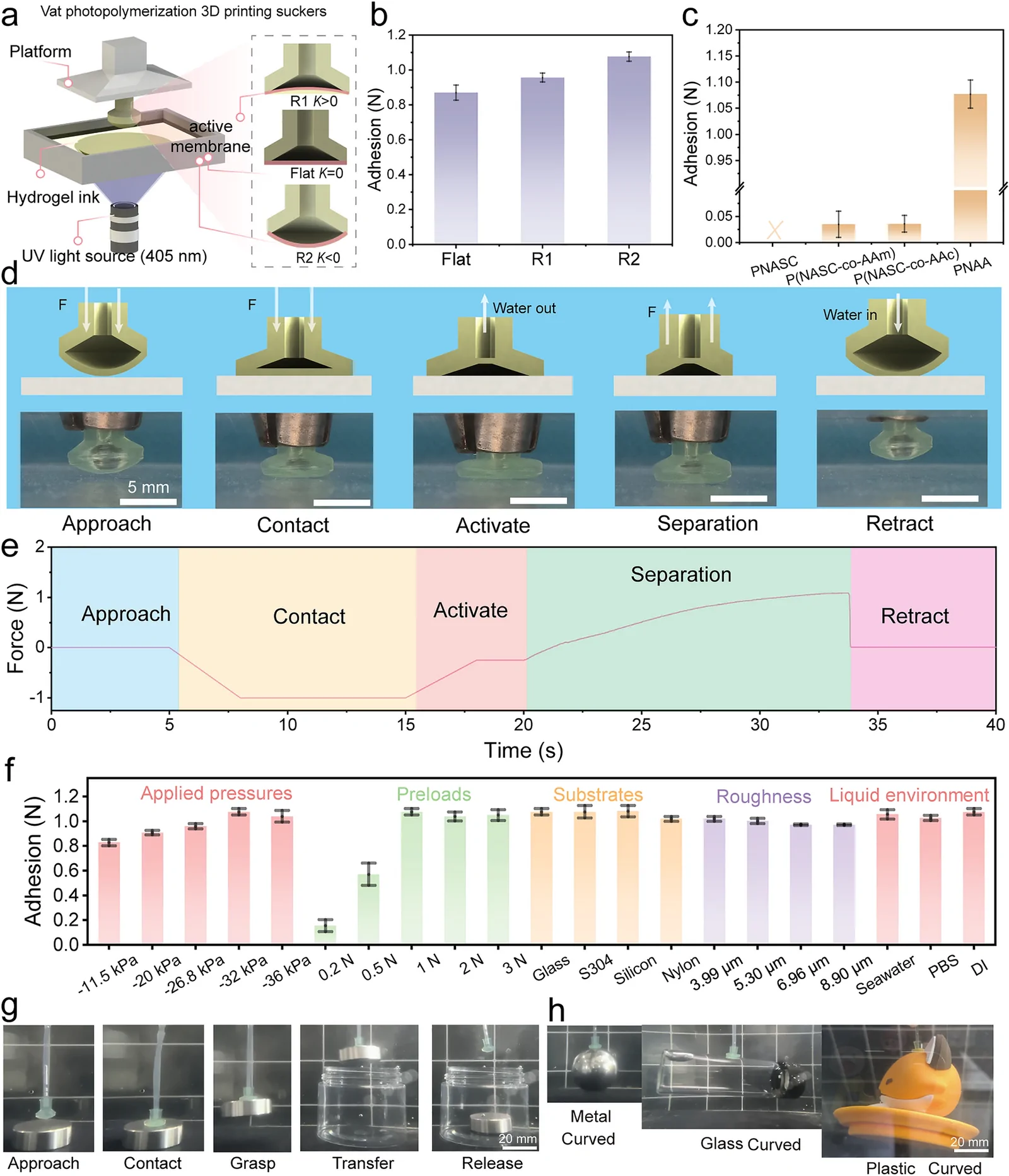
Switchable adhesion performance of supramolecular hydrogel suckers. **a** Schematic sketch of the construction of hydrogel suckers (diameter 10 mm) with different curvature membranes by vat photopolymerization 3D printing (*R*1: *K* > 0, Flat: *K* = 0, and *R*2: *K* < 0). **b** Adhesion strength of the hydrogel suckers with different curvature membranes. **c** Adhesion strength of octopus-inspired suckers constructed from PNASC, PNASC-co-AAc, PNASC-co-AAm, and PNAA hydrogels. **d** Schematic showing the adhesion and pull-off procedures and evolution of hydrogel suckers cross section in different contact interface states. **e** The process of attach-and-releasing a hydrogel sucker on the surface. **f** Adhesion performance of octopus-inspired hydrogel suckers on surfaces with different applied pressures, preloads, substrates, and roughness. **g** Demonstration of the adhesion and fall off process of a stainless-steel block in an aqueous environment with a hydrogel sucker. **h** Demonstration of hydrogel sucker grasping objects with various shapes and characteristics under water conditions, including stainless steel with flat and curved surfaces, as well as glass and plastic with curved surfaces. Error bars represent ± SD (*n* = 3)

As illustrated in Fig. 3a, the octopus-inspired switchable hydrogel sucker (7 mm diameter) consists of an active membrane (red) with controlled curvature and an architecture stalk (yellow). And the sucker features a curvature on the contact surface for reinforced control of contact formation, while the angle of inclination of the outer stalk improves compliance or pullout force. The switchable adhesion mechanism of this unique suction cup structure is to drive the object attached to the active membrane by applying a pressure differential (∆*P*) within the stalk cavity and achieve a controlled release, defined as ∆*P* = Pinput − Pambient. To wit, the application of negative pressure (∆*P* < 0) favors adhesion to grasp the object, while neutral pressure (∆*P* = 0) causes the object to release autonomously. Additionally, the manipulation of the input pressure allows for precise control over adhesion strength of the suction cup structure. Octopus-inspired hydrogel suckers with different curvature structures (*R*1: *K* > 0, Flat: *K* = 0, and *R*2: *K* < 0) have exceptional adhesion properties (Fig. 3b). It is well known that one of the main contributions of suckers to adhesion is the utilization of suction by creating interfacial pressure between the structural cavity and the substrate. As revealed in Fig. 3c, it was found that the sucker structures constructed by PNASC, P(NASC-co-AAc), and P(NASC-co-AAm) hydrogels exhibited significantly low adhesion forces during the adhesion process, which is difficult to establish reliable contact due to the discrepancies in the material stiffness. In contrast, PNAA hydrogel sucker with the best mechanical properties is able to achieve greatly superior adhesion strength. In addition, the surface wetness has an important influence on the adhesion performance of sucker. As shown in Fig. S8, the presence of hydrophilic groups, such as carboxylate and amide groups, results in an overall hydrophilic state of the hydrogel's surface. The incorporation of AAc into the NASC monomer increased the water contact angle from approximately 25° to 60°. This change is primarily attributed to the strong hydrogen bonding between NASC and AAc, which facilitates pronounced microphase separation and the formation of a densified network structure. Conversely, the introduction of acrylamide results in a substantial number of weak hydrogen bonds, leading to a looser network and consequently enhancing the hydrogel's hydrophilicity. A key challenge for an ideal suction cup structure is to be able to generate stable interfacial pressures for both attachment and release while minimizing damage to the surface of the attached object. Consequently, the movement of the variable curvature active membrane can be precisely regulated by controlling the differential pressure, thereupon promoting gentle and reliable attachment to irregular surfaces during the adhesion process (Fig. 3d). As illustrated in Fig. 3e, the octopus-inspired hydrogel suckers demonstrated adhesion (1.08 ± 0.03 N) and can be accurately manipulated by altering the differential pressure and the preload force. Furthermore, these octopus-inspired hydrogel suckers can adhere to various substrates, including silicone, glass, S304 stainless steel, and nylon. Surface roughness is a critical factor influencing interfacial contact, and different surface roughness levels correspond to the preparation processes described in previous work [16]. It is clear that the adhesion of the hydrogel suckers depends largely on the surface roughness of the substrate (Figs. 3f and S9). Furthermore, the dimensional shrinkage rate of the hydrogel remains essentially unchanged after immersion in seawater for 7 days, further confirming the stability of the hydrogel suction cups (Fig. S10). Based on the outstanding switchable adhesion capability and environment adaptability of the octopus-inspired hydrogel suckers, we further explored their practical applications in manipulation such as grasping and releasing underwater objects. As shown in Fig. 3g, h, the hydrogel suckers are able to easily grasp and release objects with various shapes and characteristics under water conditions, including stainless steel with flat and curved surfaces, as well as glass and plastic with curved surfaces (Videos S1).

### Fabrication and Performance Characterization of Hydraulically Driven Hydrogel Soft Actuators

**Fig. 4:**
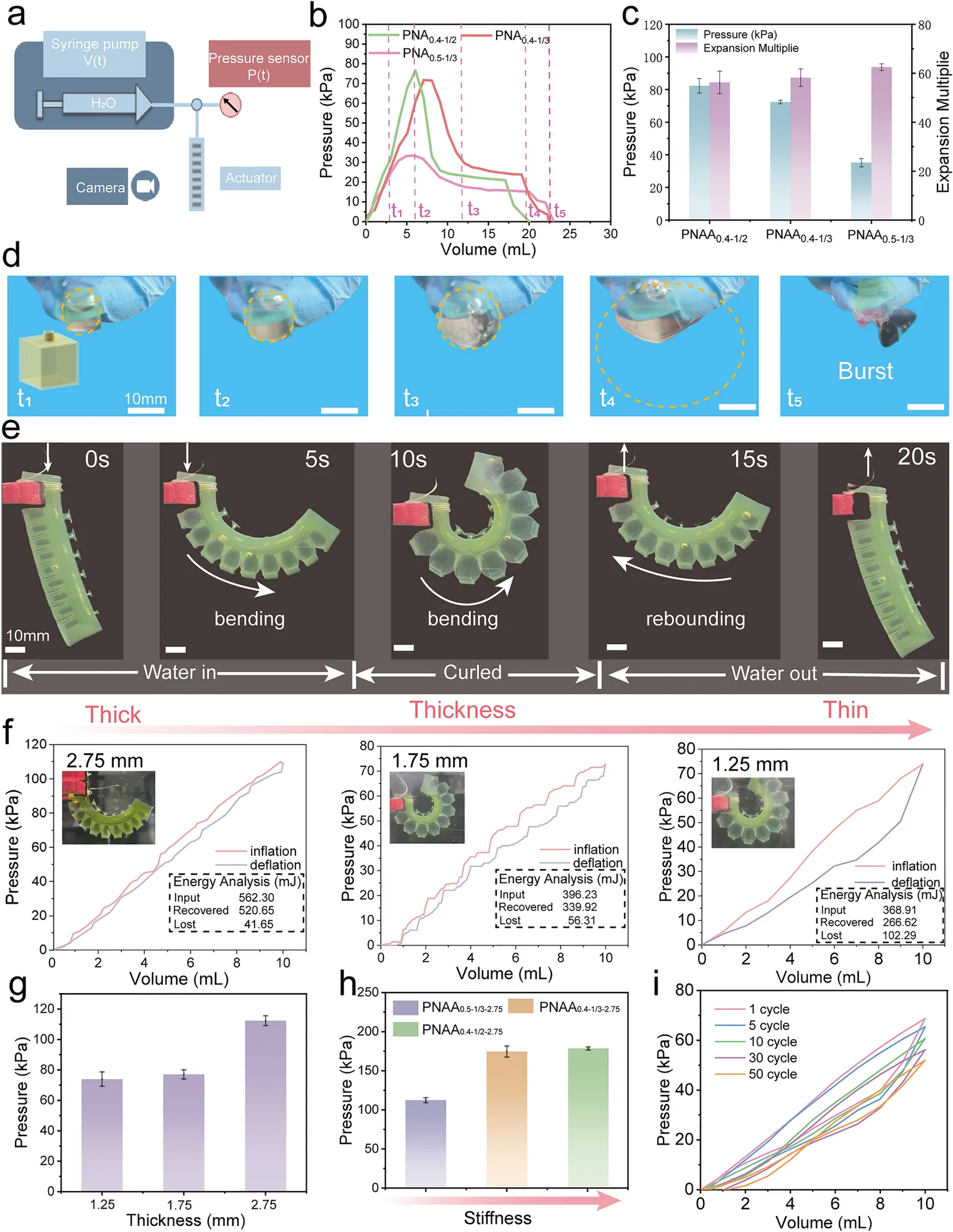
Fabrication and performance characterization of hydraulically driven hydrogel soft actuators. **a** Schematic diagram of an experimental system for measuring the driven performance of a hydrogel soft actuator. **b** Pressure versus volume plots of a cuboid-shaped hydraulic device with three different stiffnesses during expansion. **c** The expansion multiple and the corresponding pressure required to drive hydrogel hydraulic devices with three stiffnesses. **d** Photographs of a hydrogel hydraulic device with a cuboid shape under different expansion states. **e** Photographs of a hydrogel hydraulic drive in the state of expansion, bending and rebound. **f** Pressure versus volume curves of hydraulically driven hydrogel soft actuators with miscellaneous thicknesses. The inserts show the bending states of soft actuators at the same pressure. **g**–**h** The driving pressures required for the different thicknesses and stiffness hydrogel actuators obtained from a 10 mL input volume. **i** Stability of supramolecular hydrogel soft actuator with a thickness of 1.25 mm over 50 consecutive cycles. Error bars represent ± SD (*n* = 3)

To evaluate the performance of hydraulically driven hydrogel soft actuators, the designed hydrogel actuators were hydraulically inflated and deflated by means of a syringe pump and real-time data acquisition using a pressure sensor and a digital camera (Fig. 4a). As revealed in Fig. 4b–d, we first measured the expansion multiplier and the required pressure for a cuboid hydraulic device with dimensions of 7 mm × 7 mm × 7 mm and a thickness of 0.7 mm. Cubic pneumatic devices with a cavity volume of 0.343 mL fabricated from PNAA hydrogels with three different stiffnesses showed a similar process in volume expansion after water filling. Besides, the pressure test curves initially exhibited a significant increase with the rising water injection amount during the initial to *t*_2_ stage (Fig. 4b). However, the volume change of the hydraulic cubic pneumatic devices at this stage is very small (Fig. 4d), mainly due to the superior elastic properties of this supramolecular hydrogel that prevent it from undergoing significant deformation. Subsequently, the pressure gradually decreases to a steady state (i.e., from *t*_2_ to *t*_3_), and the elastic phase of the hydrogel progressively shifts to the plastic phase due to the gradual destruction of the molecular chains. In the t_3_ to t_4_ stage thereafter, the hydrogel hydraulic cubic pneumatic devices expand rapidly under steady pressure (Fig. 4b, d). The hydrogel hydraulic cubic pneumatic device ruptures until the t_5_ stage. Based on this principle, it is clearly seen that the hydraulic cubic pneumatic devices of PNAA supramolecular hydrogels with different stiffness (i.e., PNAA_0.4–1/2_, PNAA_0.4–1/3_, and PNAA_0.5–1/3_) showcased exceptional expansion performance, and achieved more than 60 times the expansion effect (Fig. 4c). It is worth mentioning that the minimum actuation pressure required for a hydraulic cubic pneumatic device made of a lower modulus PNAA_0.5–1/3_ hydrogel is only 35.18 ± 2.53 kPa. Furthermore, we fabricated several intricate hydraulic hydrogel actuators with alterable thicknesses and stiffness, as well as estimating their required drive pressures. It can be seen that the initially straight hydrogel actuator bends inward as water is introduced into the actuation chambers and then forms a complete circle, and the actuator swiftly returns to a straight configuration when the water is discharged (Fig. 4e and Video S2). What is more, it would be said that the hydrogel soft actuators with high stiffness and thicknesses were only capable of deforming into a semicircular shape within the same timeframe and even exhibit a smaller bending deformation (Fig. S11). To quantify the fluid transport behavior during the actuation, as illustrated in Fig. 4f–h, we determined the pressure versus volume plots of the PNAA hydrogel soft actuators with multiple thicknesses when submerged in water to counteract gravitational effects. It was found that PNAA hydrogel actuators with smaller wall thickness demonstrated reduced pressure, energy, and dissipated energy requirements at the same stiffness, thereupon favoring the capacity of soft actuator to bend completely (Fig. 4f, g). Additionally, the softer PNAA hydrogel hydraulic actuator at the same wall thickness (2.75 mm) requires a lower actuation pressure (Fig. 4h). This remarkable comparison clearly underscores that supramolecular hydrogel soft hydraulic actuators with lower modulus and wall thickness offer superior drive performance. Furthermore, we have included a comparison of a gripper made from hydrophobic silicon-based materials to highlight the advantages of our hydrogel gripper (Figs. S12–14) [47]. The hydrogel gripper demonstrates exceptional driving performance characterized by low driving pressure (76 kPa), superior deformation angles (150°), and high gripping force (1.5 N). In various liquid environments, including seawater, deionized water, and phosphate-buffered saline (PBS) solution, the hydrogel gripper demonstrates consistent performance (Fig. S15). More importantly, the hydraulic hydrogel soft actuator successfully endures 50 continuous actuation cycles without experiencing failure or leakage (Fig. 4i), further emphasizing its distinguished robustness and functionality.

### Octopus-Inspired Hydrogel Grippers for Underwater Gripping and Manipulation

**Fig. 5:**
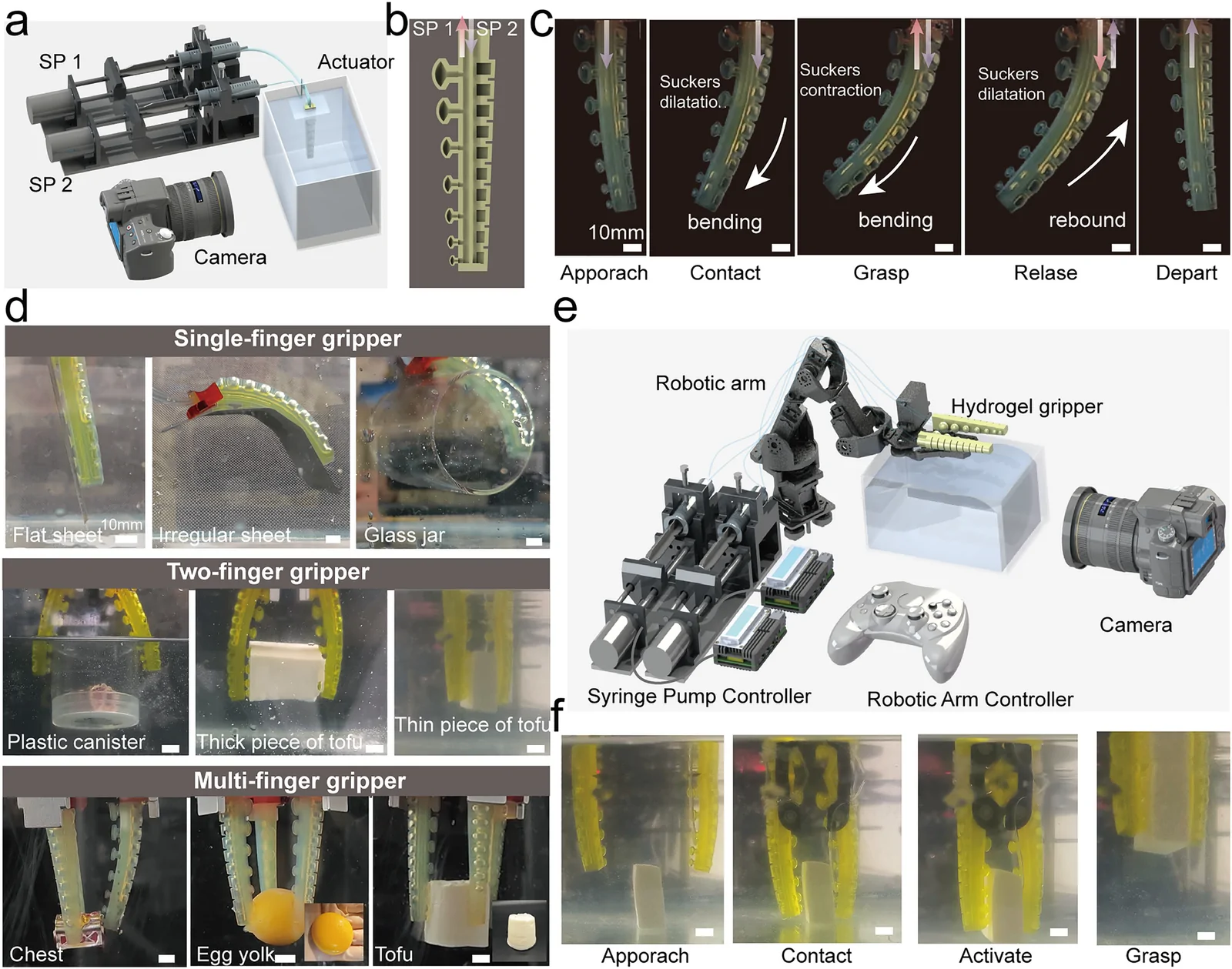
Versatile demonstration of the octopus-inspired hydrogel soft grippers for underwater gripping and manipulation. **a** Schematic illustration of an experimental system used to observe the operation of a hydrogel soft gripper. **b** Schematic showing an octopus-inspired hydrogel soft gripper consisting of a pneumatic chamber connected to suckers and a flexible pneumatic channel. **c** Photograph of a hydrogel soft gripper at different stages of bending and rebounding. **d** Designed and manufactured single-finger, two-finger, and multi-finger hydrogel soft grippers for handling objects of different shapes (spheres, cylinders, and cubes), surfaces (flat, curved, and folded) and stiffness attributes (stainless steel, glass, plastic, and ultra-soft foods like tofu and egg yolks). **e** Schematic sketch of a robotic arm equipped with hydrogel grippers for the maneuvering of underwater objects. **f** A robotic arm equipped with a hydrogel gripper is capable of nondestructively gripping and releasing ultra-soft tofu blocks underwater

Based on the soft and tough mechanical properties of the supramolecular hydrogels, the adaptive adhesion characteristics of the suckers, and the excellent hydraulic actuation performance of the soft actuators, as depicted in Fig. 5a, thereupon we contrived and developed an octopus-inspired hydraulically actuated hydrogel soft grasper, which is controlled independently by two syringes. The octopus-inspired hydrogel soft gripper consists of two separate tubes connecting the suckers and tentacles (Fig. 5b), one of which is connected to the pneumatic chamber of the suckers (denoted as SP 1), while the other serves as a flexible pneumatic channel for operating the tentacles (denoted as SP 2). As shown in Fig. 5c, SP 2 initially injects a specific volume of water into the tentacle at a controlled speed, causing the tentacle to bend. Meanwhile, SP 1 activates to expel water outward after the tentacle approaches the object, which causes the suckers on the tentacles to retract to achieve switchable adhesion. Subsequently, the SP 2 discharges water outward to bend the tentacles backward, while SP 1 is utilized to inject water into the sucker pipe to achieve desorption release. To further prove the versatility of the octopus-inspired hydrogel grippers in handling different types of underwater objects, as shown in Fig. 5d, we designed and manufactured single-finger, two-finger, and multi-finger hydrogel soft grippers (Figs. S16 and S17). In the case of single-finger grippers, due to the adaptability of the tentacles and the adhesion of the suckers, they can achieve tight adhesion to flat, curved, and folded thin objects with irregular shapes such as stainless steel, glass, and plastic, which is hard to achieve with other single grippers. Besides, the two-finger grippers possess the capacity to manipulate some hard and ultra-soft objects with curved surface features, such as plastic bottles and soft tofu blocks of different thicknesses. More interestingly and meaningfully, the multi-finger grippers are capable of adapting to the precise gripping of complex, ultra-soft objects and enable a damage-free release of surfaces, such as egg yolks and tofu (Fig. S18, Video S3). The cooperative maneuver of the hydrogel soft gripper, which incorporates tentacle gripping force and sucker adhesion, makes it feasible to precisely grip and nondestructively release some ultra-soft and irregular objects. To realize the intelligent operation and application of hydrogel soft gripper, a robotic arm equipped with two hydrogel grippers is designed to perform some gripping operations remotely, as shown in Fig. 5d. After the remote-controlled manipulator approaches the tofu, a robotic arm equipped with hydrogel grippers is capable of nondestructively gripping and transferring ultra-soft tofu blocks underwater (Figs. 5e and S19, Video S4). The silicone gripper may potentially damage ultra-soft objects during the grasping process due to the challenges associated with establishing gentle and stable contact with these objects (Fig. S20, Video S5). The multi-finger gripper is capable of stably lifting a maximum weight of 102 g (Fig. S21). This demonstration underscored the potential applications of the coupling of the hydrogel soft gripper with the robotic arms in the transshipment and manipulation of underwater objects.

### Attachment and Maneuvering of Robots or Vehicles Equipped with Hydrogel Suckers in Challenging Underwater Conditions

**Fig. 6:**
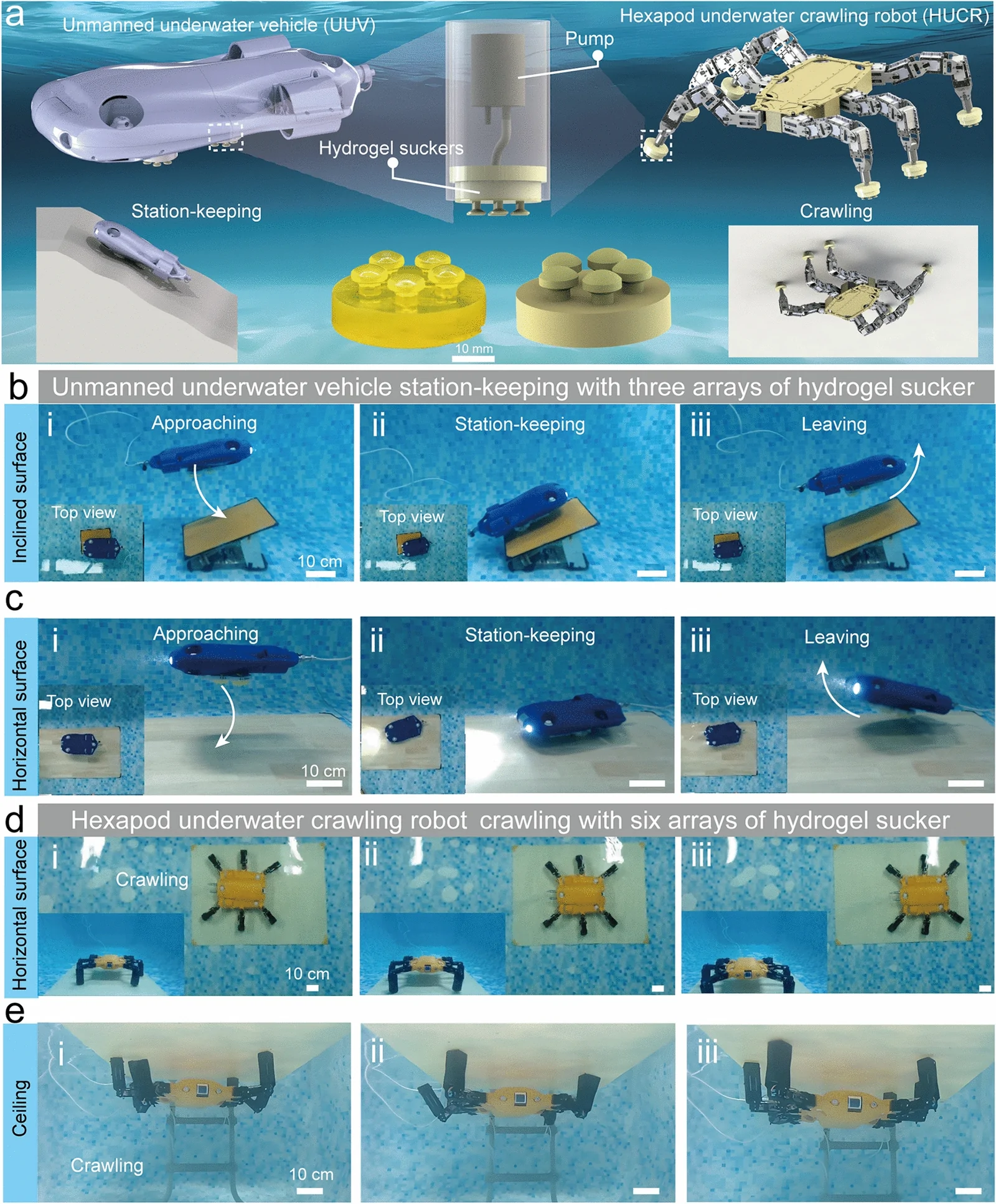
Conceptual application demonstration for underwater attachment and maneuvering of robots or vehicles equipped with hydrogel suckers. **a** Schematic diagram of an underwater unmanned vehicle and hexapod crawling robot equipped with an array of octopus-inspired hydrogel suckers. **b** Photographs of an unmanned underwater vehicle with three arrays of hydrogel sucker approaching, station-keeping, and leaving on an inclined plank. **c** Photographs of an unmanned underwater vehicle with three arrays of hydrogel sucker approaching, station-keeping, and leaving on a horizontal plank. **d**–**e** Photographs of a hexapod robot with six arrays of hydrogel sucker crawling and moving underwater

To comprehensively showcase the promising applications and multifunctional operation capabilities of the octopus-inspired hydrogel suckers in underwater environments, as revealed in Fig. 6a, we designed two classes of unmanned vehicles and hexapod crawling robots equipped with hydrogel suckers. For an unmanned underwater vehicle (UUV) with precision station-keeping and adaptive crawling maneuvers, three hydrogel adhesion sucker array structural modules were integrated into the ventral surface of the unmanned underwater vehicle (Fig. 6b, c, Video S6). It was found that this unmanned underwater vehicle could achieve approaching, attachment, crawling, and departure operations on the surface of the inclined wooden plank underwater through hydrogel sucker structures (Fig. 6b). Meanwhile, an unmanned underwater vehicle with three arrays of hydrogel sucker is also capable of approaching, station-keeping, and leaving on a horizontal plank (Fig. 6c). This result shows that this integration enables pump-regulated, adhesion-based station-keeping, and motor-driven object transport capabilities. Furthermore, we implemented a hexapod underwater crawling robot (HUCR) in which each plantar contains an array of the octopus-inspired hydrogel sucker. And this hexapod robot is able to crawl and move well on the flat plank and ceiling underwater (Fig. 6d, e, Videos S7 and S8). This proof-of-concept demonstration showcases the potential applications of the octopus-inspired hydrogel adhesive devices for many underwater intervention tasks, such as marine equipment maintenance, underwater archeology, and deep-sea exploration.

## Conclusions

This work demonstrates an octopus-inspired adaptive hydrogel soft gripper that is capable of manipulating underwater ultra-soft objects through the cooperative integration of switchable bionic suckers and hydraulically actuated tentacles. A supramolecular hydrogel with tunable mechanical properties was engineered for adaptive soft gripper fabrication through the cooperative interactions of strong and weak H-bonding. Meanwhile, vat photopolymerization 3D printing was utilized to construct an octopus-inspired hydrogel sucker that is composed of a tunable curvature membrane, a negative pressure cavity, and a pneumatic chamber, in which the hydrogel curvature membrane regulated the deadhesion of the suction cups. Furthermore, several intricate hydraulic hydrogel soft actuators with alterable thicknesses and stiffness were contrived and fabricated to optimally evaluate their expansion factor and bending deformation capacity underwater. The experimental results indicate that the octopus-inspired single-finger, two-finger, and multi-finger hydrogel soft gripper is capable of delicately handling underwater objects of diverse sizes, shapes, and attributes, and is even capable of gripping super-soft tofu and raw egg yolks with a nondestructive release of the surface. Last but not least, two classes of multifunctional underwater unmanned vehicles and hexapod crawling robots equipped with hydrogel suckers were also designed, enabling robots to manipulate objects, adhesion-based station-keeping, and crawling underwater. Overall, we anticipate these results as well as structural designs can advance underwater switchable adhesion of slippery and soft materials, which is to be broadly applicable for diverse applications spanning robotic manipulation, biomedical engineering, and deep-sea exploration.

## Supplementary Information

Below is the link to the electronic supplementary material.

Supplementary file 1 (MP4 4773 KB)

Supplementary file 2 (MP4 2652 KB)

Supplementary file 3 (MP4 9723 KB)

Supplementary file 4 (MP4 2703 KB)

Supplementary file 5 (MP4 3193 KB)

Supplementary file 6 (MP4 5824 KB)

Supplementary file 7 (MP4 2413 KB)

Supplementary file 8 (MP4 2400 KB)

Supplementary file 9 (DOCX 12768 KB)
