# Supplementary material for: Octopus-Inspired Self-Adaptive Hydrogel Gripper Capable of Manipulating Ultra-Soft Objects
Source: Nanomicro Lett. 2025 Aug 19;18:33. doi: 10.1007/s40820-025-01880-4 (PMC12364788; doi:10.1007/s40820-025-01880-4)
Supplement: Supplementary file 9 — (DOCX 12768 KB) [file 40820_2025_1880_MOESM9_ESM.docx]

**Octopus-Inspired Self-Adaptive Hydrogel Gripper Capable of Manipulating Ultrasoft Objects**

Yixian Wang ^1, 2^, Desheng Liu ^1*^, Danli Hu ^1^, Chao Wang ^3^, Zonggang Li ^3^, Jiayu Wu ^1^, Pan Jiang ^1^, Xingxing Yang ^2^, Changcheng Bai ^1^, Zhongying Ji ^1^, Xin Jia ^2*^, Xiaolong Wang ^1, 2*^

^1^ State Key Laboratory of Solid Lubrication, Lanzhou Institute of Chemical Physics, Chinese Academy of Sciences, Lanzhou 730000, PR China

^2^ School of Chemistry and Chemical Engineering/State Key Laboratory Incubation Base for Green Processing of Chemical Engineering , Shihezi 832003, PR China

^3^ School of Mechanical Engineering, Lanzhou Jiaotong University, Lanzhou, 730070, PR China

*Corresponding author. E-mail: liudesheng@licp.cas.cn; jiaxin@shzu.edu.cn; wangxl@licp.cas.cn

**Table S1** Hydrogel precursor solution formulations

| Hydrogels | NASC (g) | AAc (g) | AAm (g) | LAP (g) | DMSO(g) | H_2_O(g) |
| --- | --- | --- | --- | --- | --- | --- |
| PNAA_0.4-3/1_ | 3.5714 | 1.0714 | 0.3572 | 0.025 | 10.5 | 4.5 |
| PNAA_0.4-2/1_ | 3.5714 | 0.9524 | 0.4762 | 0.025 | 10.5 | 4.5 |
| PNAA_0.4-1/1_ | 3.5714 | 0.7143 | 0.7143 | 0.025 | 10.5 | 4.5 |
| PNAA_0.4-1/2_ | 3.5714 | 0.4762 | 0.9524 | 0.025 | 10.5 | 4.5 |
| PNAA_0.4-1/3_ | 3.5714 | 0.3572 | 1.0714 | 0.025 | 10.5 | 4.5 |
| PNAA_0.5-2/1_ | 3.3333 | 1.1112 | 0.5556 | 0.025 | 10.5 | 4.5 |
| PNAA_0.5-1/1_ | 3.3333 | 0.8333 | 0.8333 | 0.025 | 10.5 | 4.5 |
| PNAA_0.5-1/2_ | 3.3333 | 0.5556 | 1.1112 | 0.025 | 10.5 | 4.5 |
| PNAA_0.5-1/3_ | 3.3333 | 0.4167 | 1.2500 | 0.025 | 10.5 | 4.5 |

Note: NASC accounts for 25% of the total mass of the ink. Among PNAA_X-Y/Z_, X represents the ratio of the total mass of AAm and AAc to the mass of NASC, and Y/Z represents the mass ratio of AAc/AAm respectively.

Chemical structure characterization

The Fourier transform infrared spectroscopy (FTIR) confirmed the molecular structure of NASC monomer. The NASC monomer revealed diagnostic absorption bands at: 3439 (NH), 3330(NH), 3225 (NH), 3056 (NH), 1685 (C=O), 1627(C=C), 1594 (NH). In addition, Figure S2 and Figure S3 present the NMR spectra of the NASC monomer. The corresponding positions have been clearly marked in the figures. In the 1H NMR spectrum, the chemical shifts are observed at δ=9.8 (Ha), 7.9 (Hb), 6.2 (Hc), 6.0 (Hd), and 5.7, 6.2 (He). Similarly, in the 13C NMR spectrum, the signals appear at δ = 164.6 (Ca, -CO-), 159.1 (Cb, -CO-), 130.1 (CH₂-CH-), and 126.7 (CH₂-CH-). These results confirm the successful synthesis of the NASC monomer. These results confirm the successful synthesis of the NASC monomer.

Double bond conversion testing: Real-time ATR-FTIR analysis was employed to track the conversion of double bonds at approximately the 810 cm−1 peak under 405 nm UV irradiation with a UV-LED light (10 mW cm−2 , 405 nm). The ester carbonyl absorption peak (C=O) at 1725 cm−1 in the FTIR spectrum can be used as an internal standard. The peak area ratio between the C=C and C=O was used to monitor the monomer conversion. Double bone conversion at a given time(t) was calculated by $\alpha=1-\left( \frac{A_{c=c}}{A_{c=o}} \right)_{t}/\left( \frac{A_{c=c}}{A_{c=o}} \right)_{t=0}$


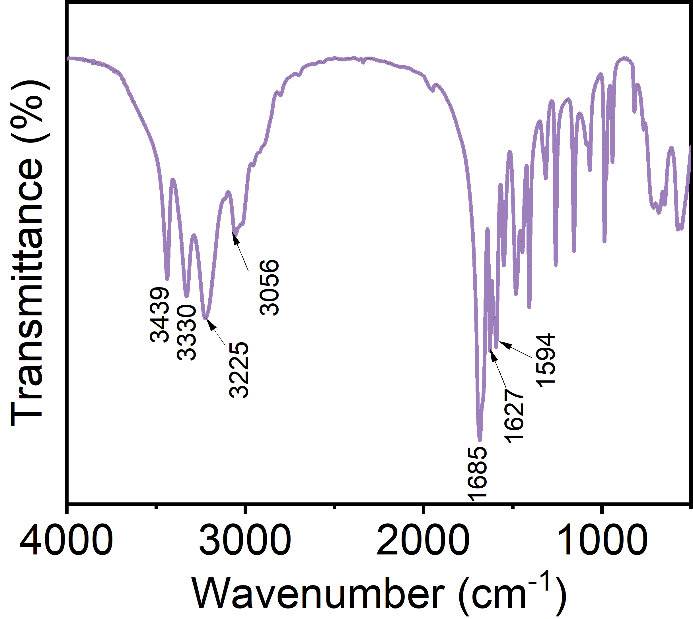


**Figure S1.** FTIR spectra of the NASC monomer.


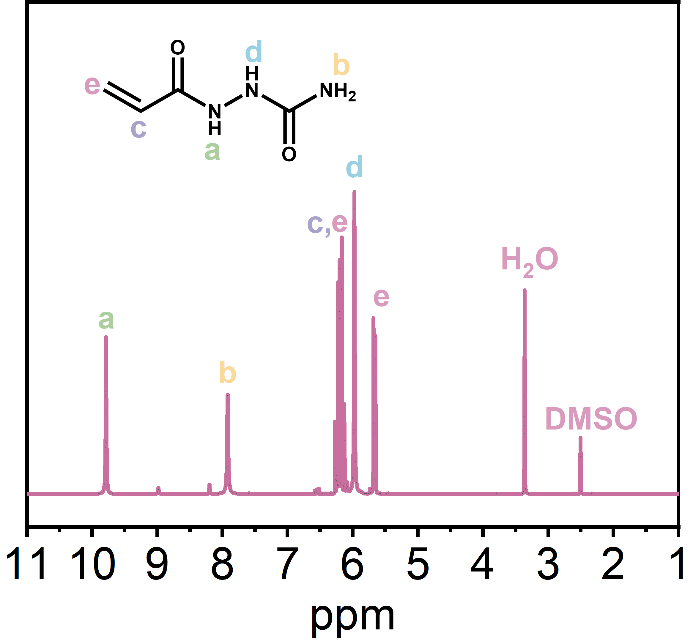


**Figure S2.** ^1^H-NMR spectra of the NASC monomer.


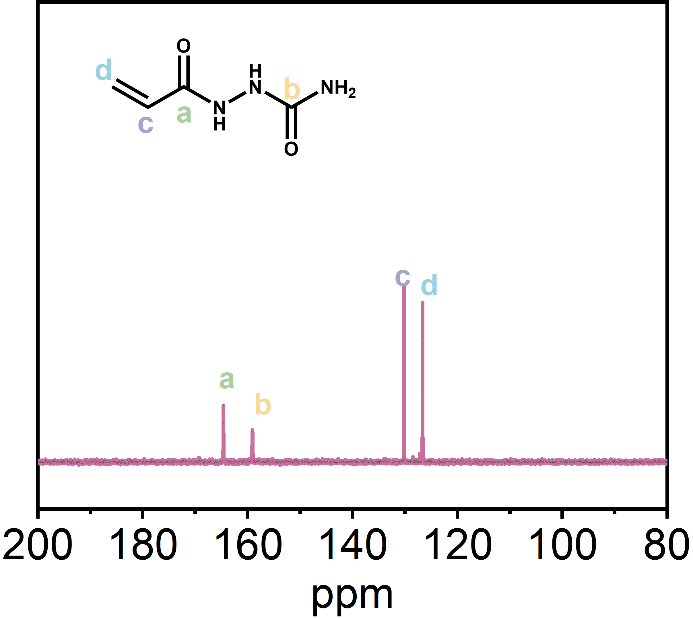


**Figure S3.** ^13^C-NMR spectra of the NASC monomer.


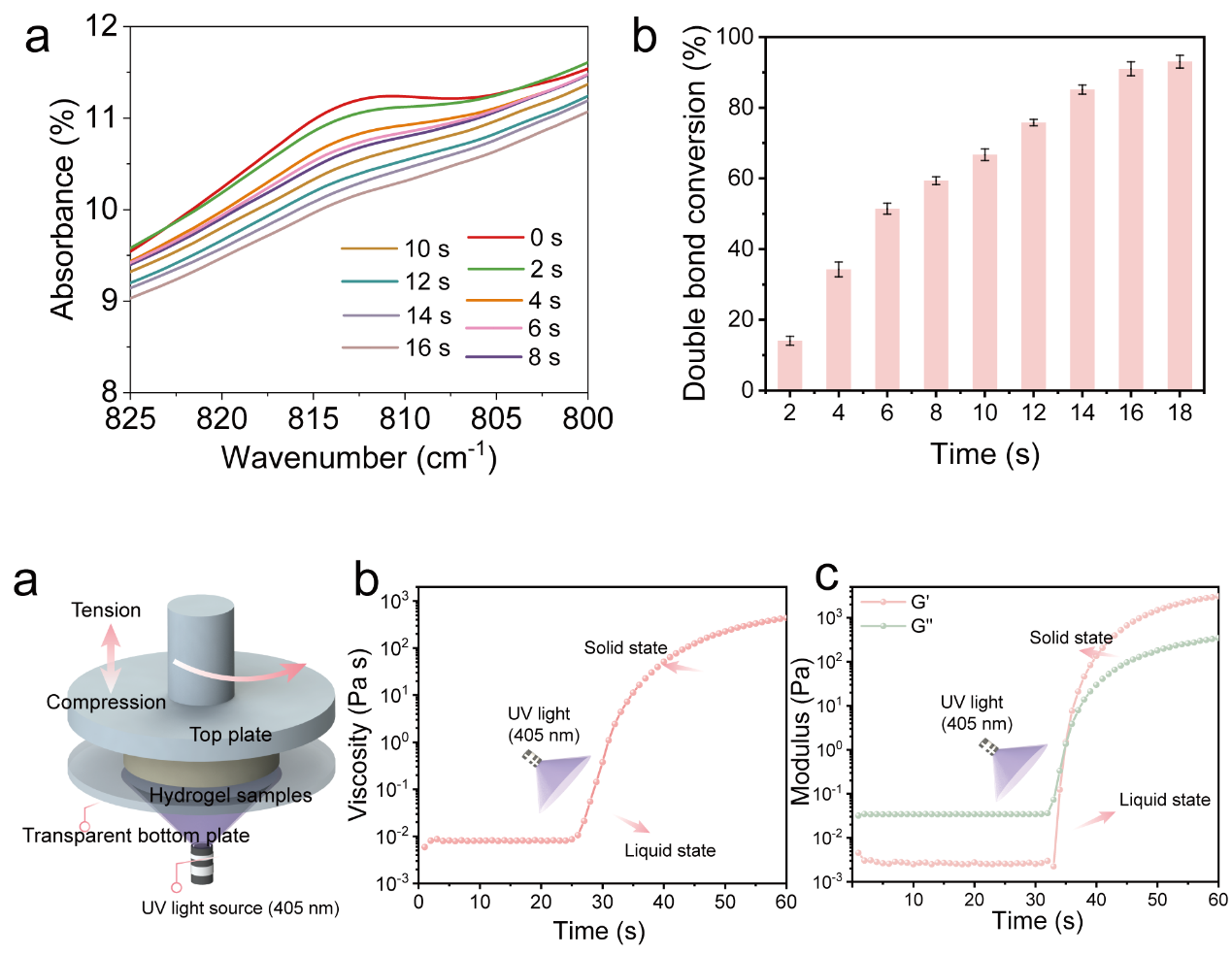


**Figure S4.** Photopolymerization rheology testing. (a) Illustration of photopolymerization rheology testing. (b) the viscosity of the photosensitive hydrogel inks varies with the UV irradiation time. (c) the modulus of the inks as a functional of the UV irradiation time.


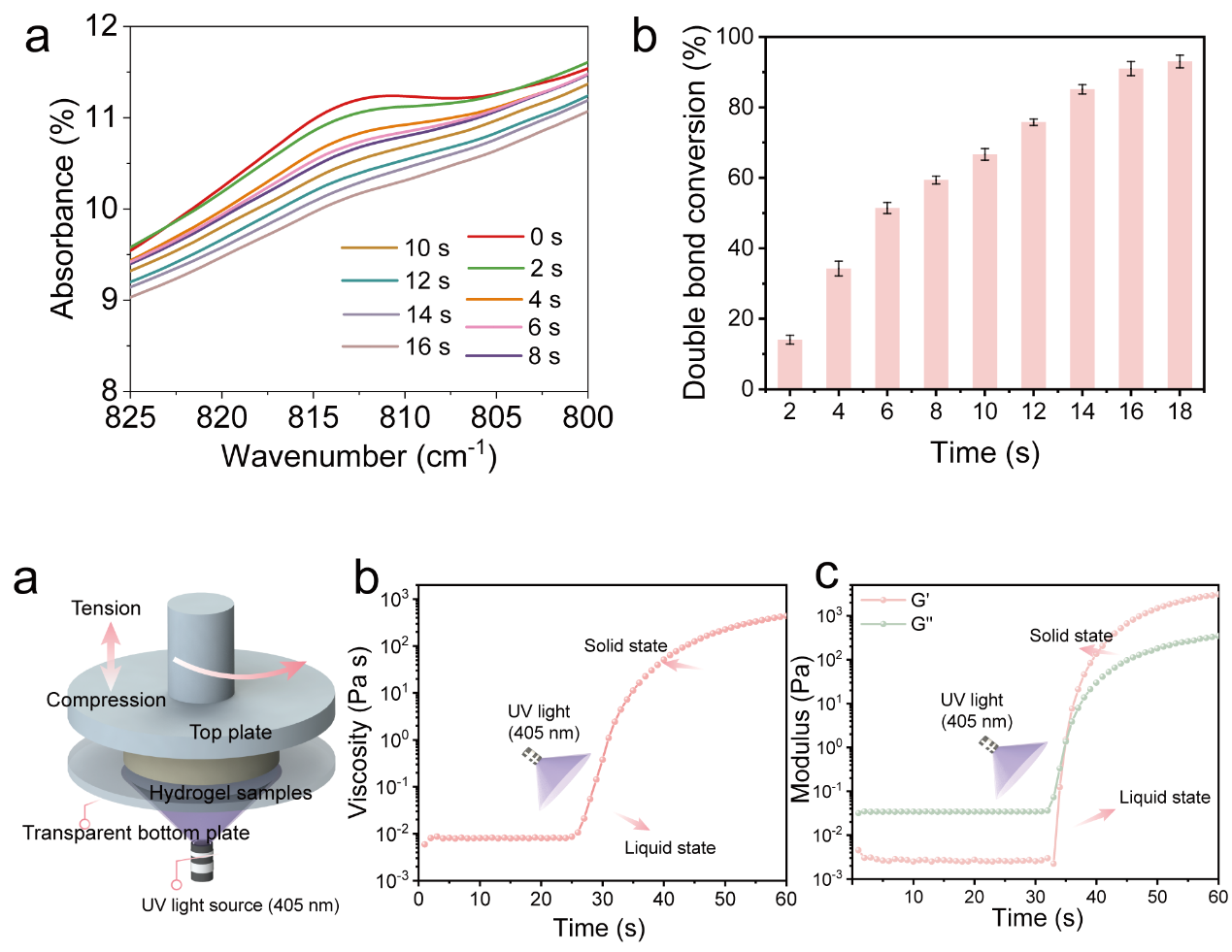


**Figure S5.** Double bond conversion testing (a) the IR spectra of hydrogels with different UV illumination times. (b) double bond conversion of hydrogel inks.


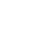

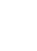

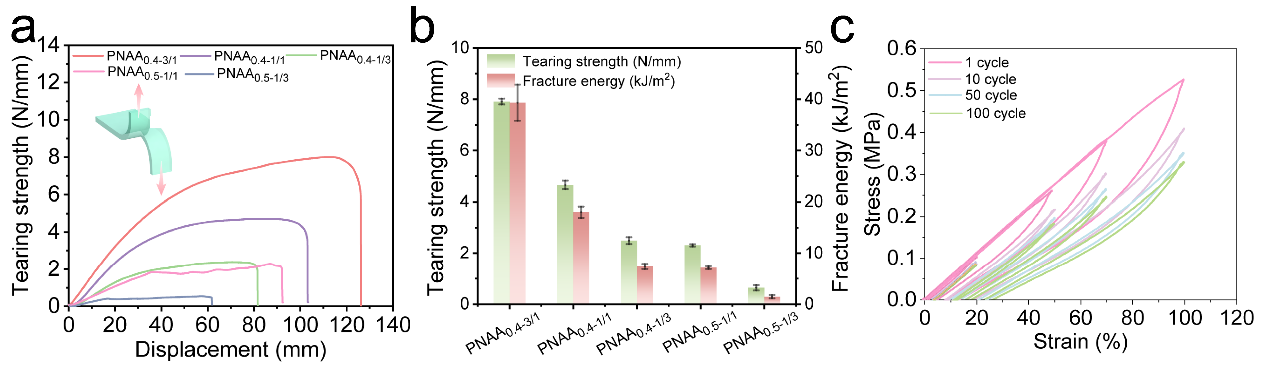


**Figure S6.** (a) Tearing strength–displacement curves of PNAA hydrogels. (b) Tearing strength and fracture energy of PNAA hydrogels. (c) Tensile loading-unloading curves of PNAA_0.5-1/3_ under 20%, 50%, 70%, 100% strains.
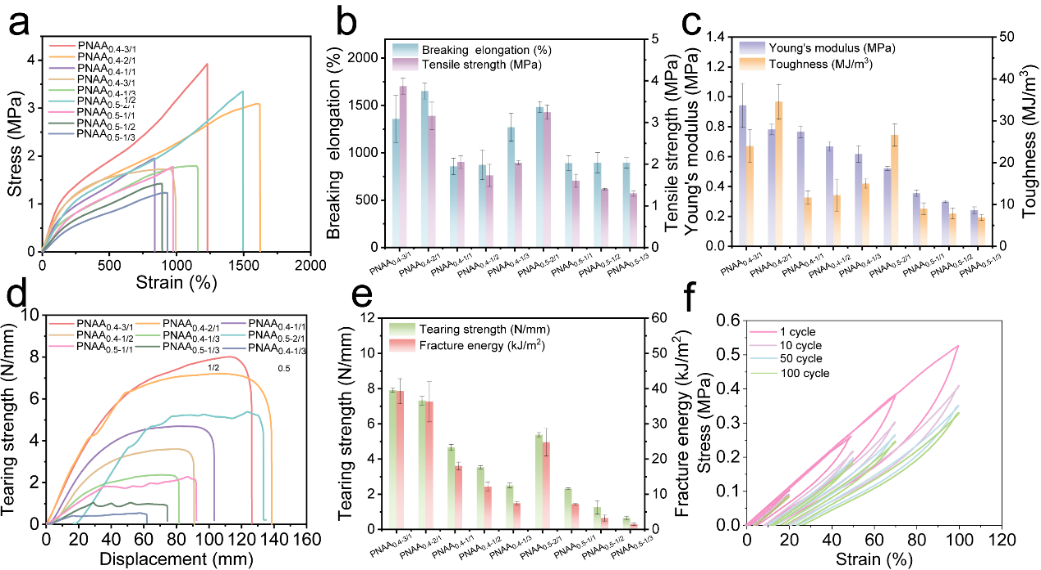


**Figure S7.** (a) Stress–strain curves of PNAA hydrogels. (b) Breaking elongation and tensile strength. (c) Young’s modulus and toughness. (d) Tearing strength–displacement curves. (e) Tearing strength and fracture energy.(f) Tensile loading-unloading curves of PNAA0.5-1/3 under 20%, 50%, 70%, 100% strains.


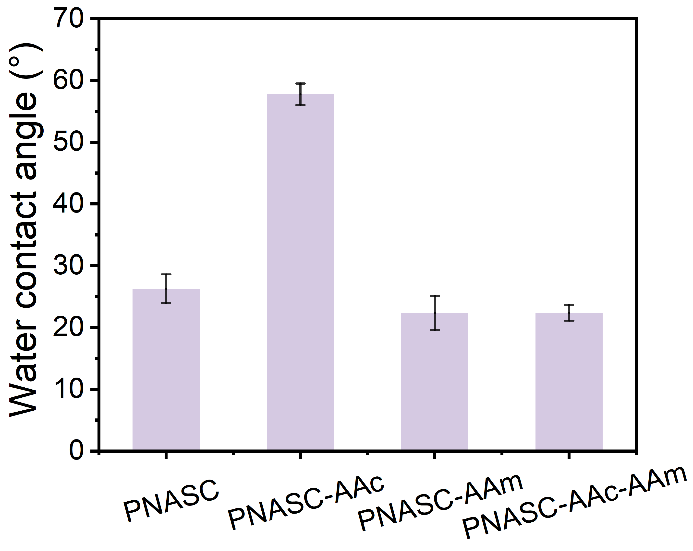


**Figure S8.** The water contact angle of hydrogels.


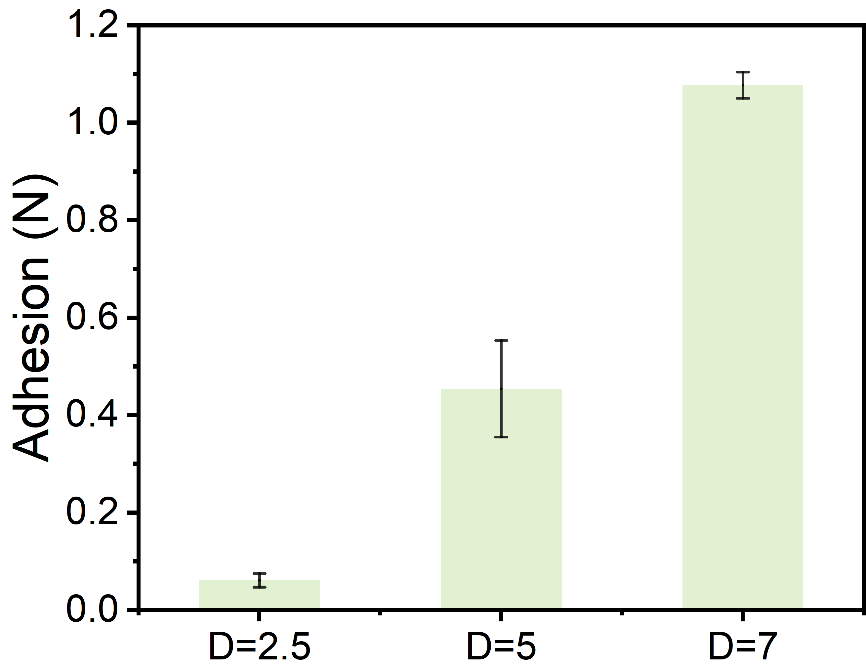


**Figure S9.** The adhesion of hydrogel suction cups with different diameters.


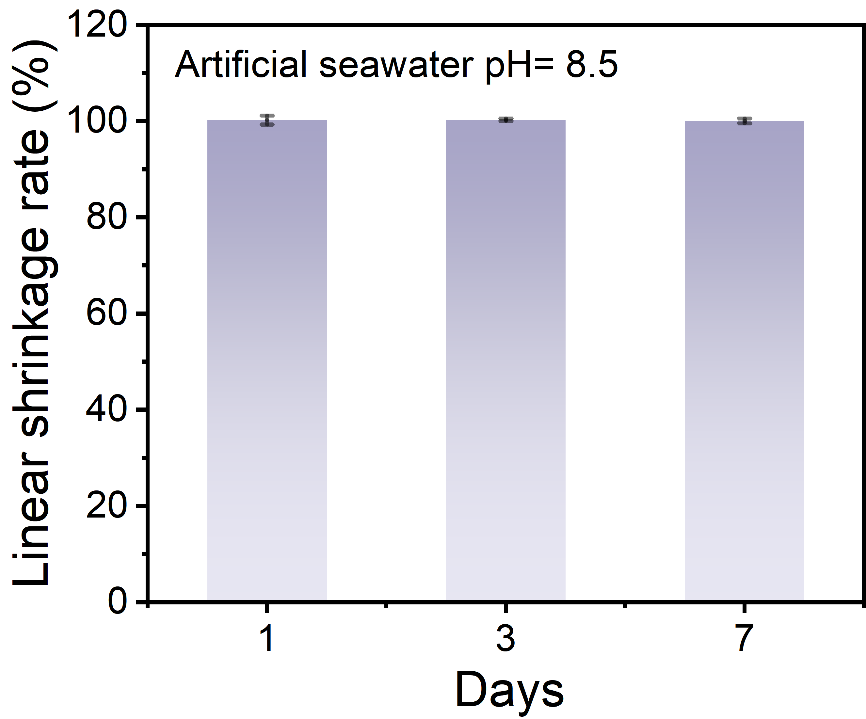


**Figure S10.** The linear shrinkage rate of hydrogel suckers under seawater


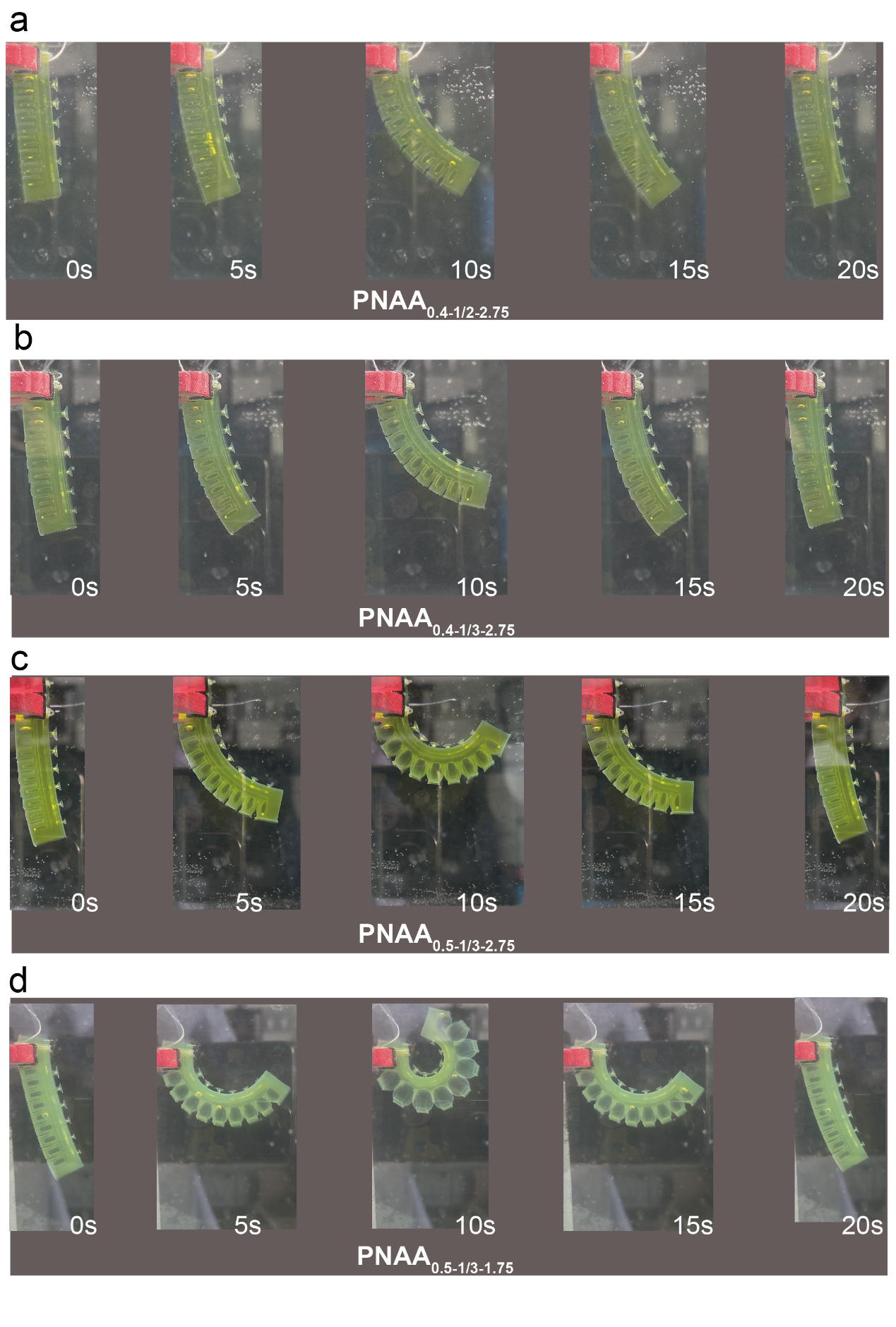
**Figure S11**. Photos of the driving process of (a) PNAA_0.4-1/2-2.75_ hydrogel actuator. (b) PNAA_0.4-1/3-2.75_ hydrogel actuator. (c) PNAA_0.5-1/3-2.75_ hydrogel actuator. (d) PNAA_0.5-1/3-1.75_ hydrogel actuator.


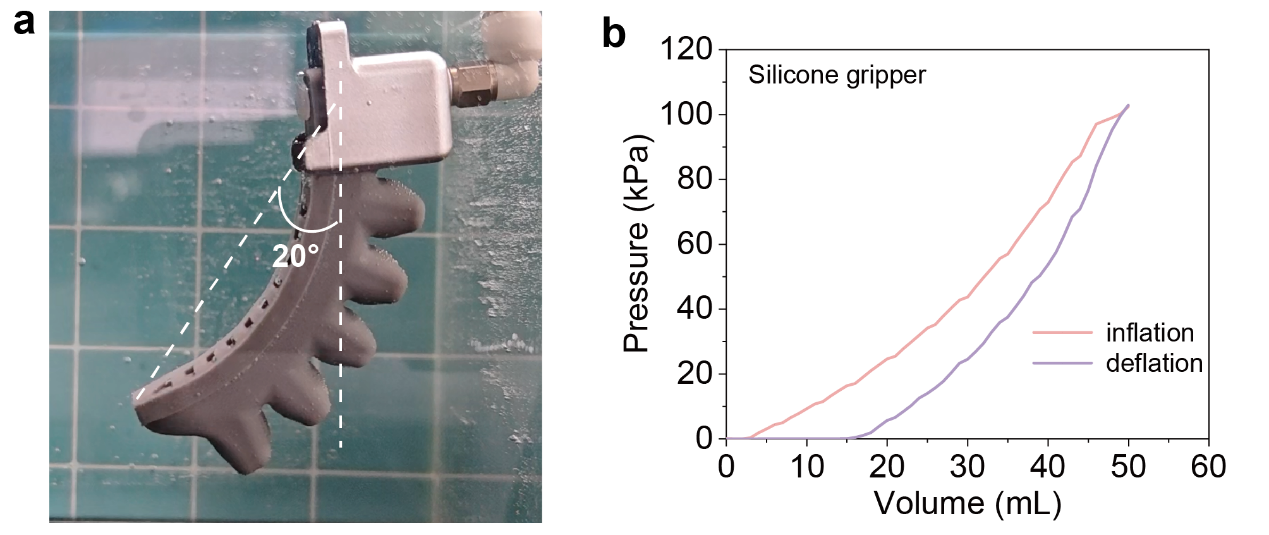


**Figure S12**. (a) The deformation angle of commercial silicone gripper with a pressure of 100kPa.(b) Pressure-volume curve of commercial silicone gripper.


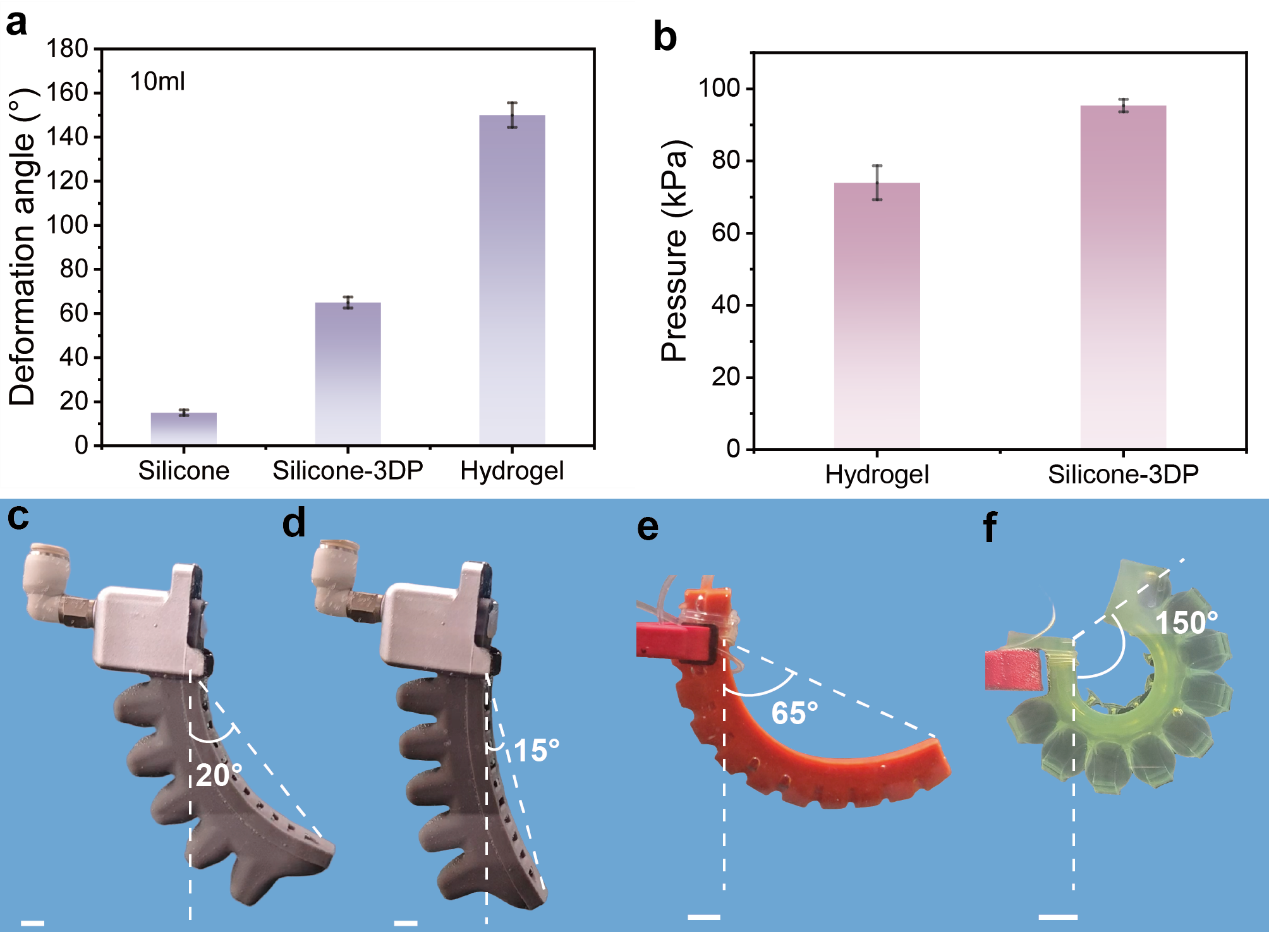


**Figure S13**. (a) The deformation angle of grippers for different materials.(b) The driving pressures required for the different materials. (c-f) Photographs of deformation thresholds for grippers made of different materials. Scale bar: 10mm.


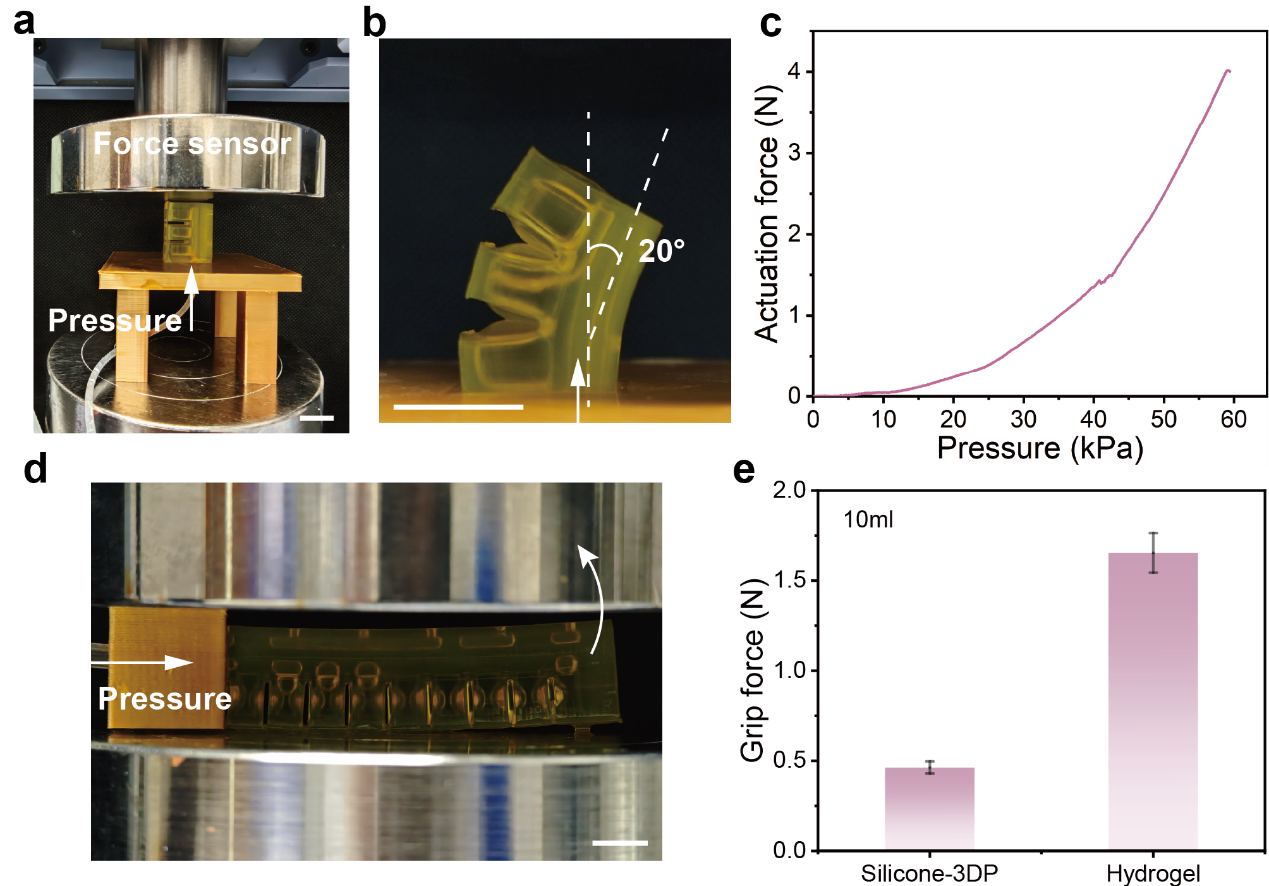


**Figure S14**. (a) Experimental setup for the actuation force measurement. (b) Photographs of deformation thresholds for unit-segment hydrogel actuator. (c) Actuation force versus pressure of unit-segment hydrogel actuator. (d) Experimental setup for the grip force measurement. (e) Grip force of grippers for different materials. Scale bar: 10mm.


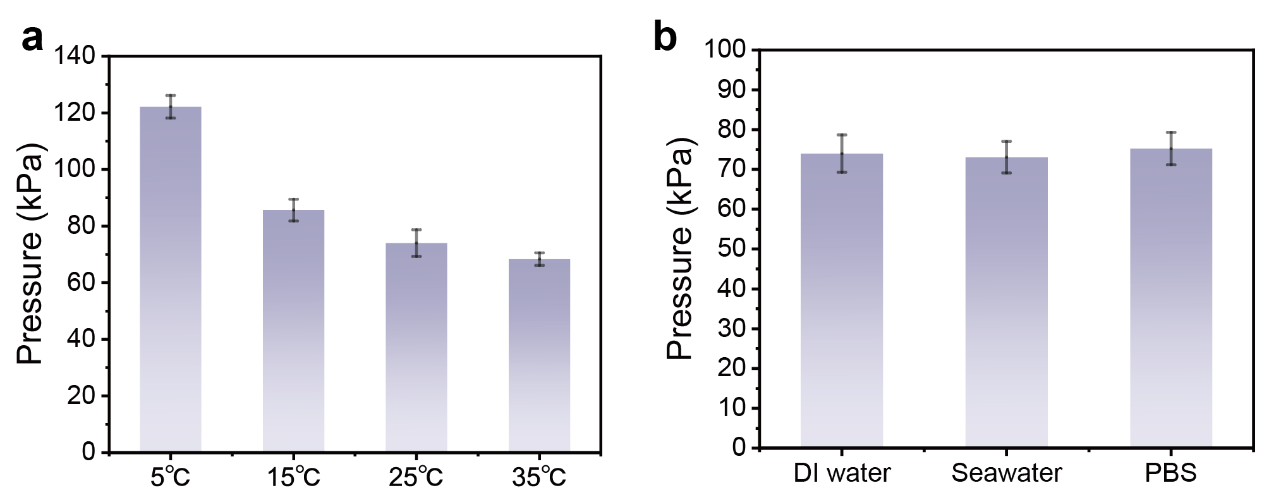


**Figure S15**. The driving pressure for hydrogel grippers to reach the deformation threshold at (a) different temperatures. (b) different environments.


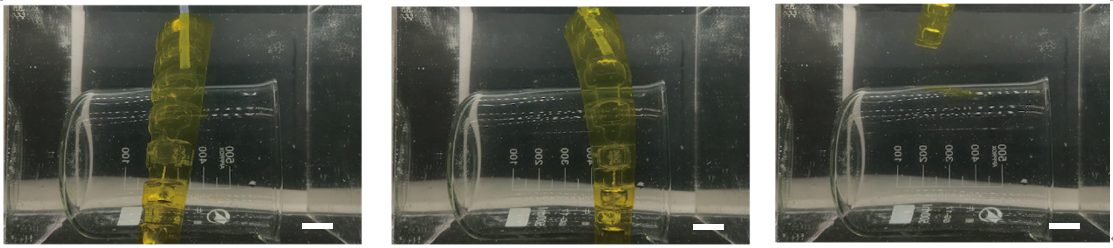


**Figure S16**. Photographs of the hydrogel gripper without suction cups failing to grasp objects. Scale bar: 10mm.


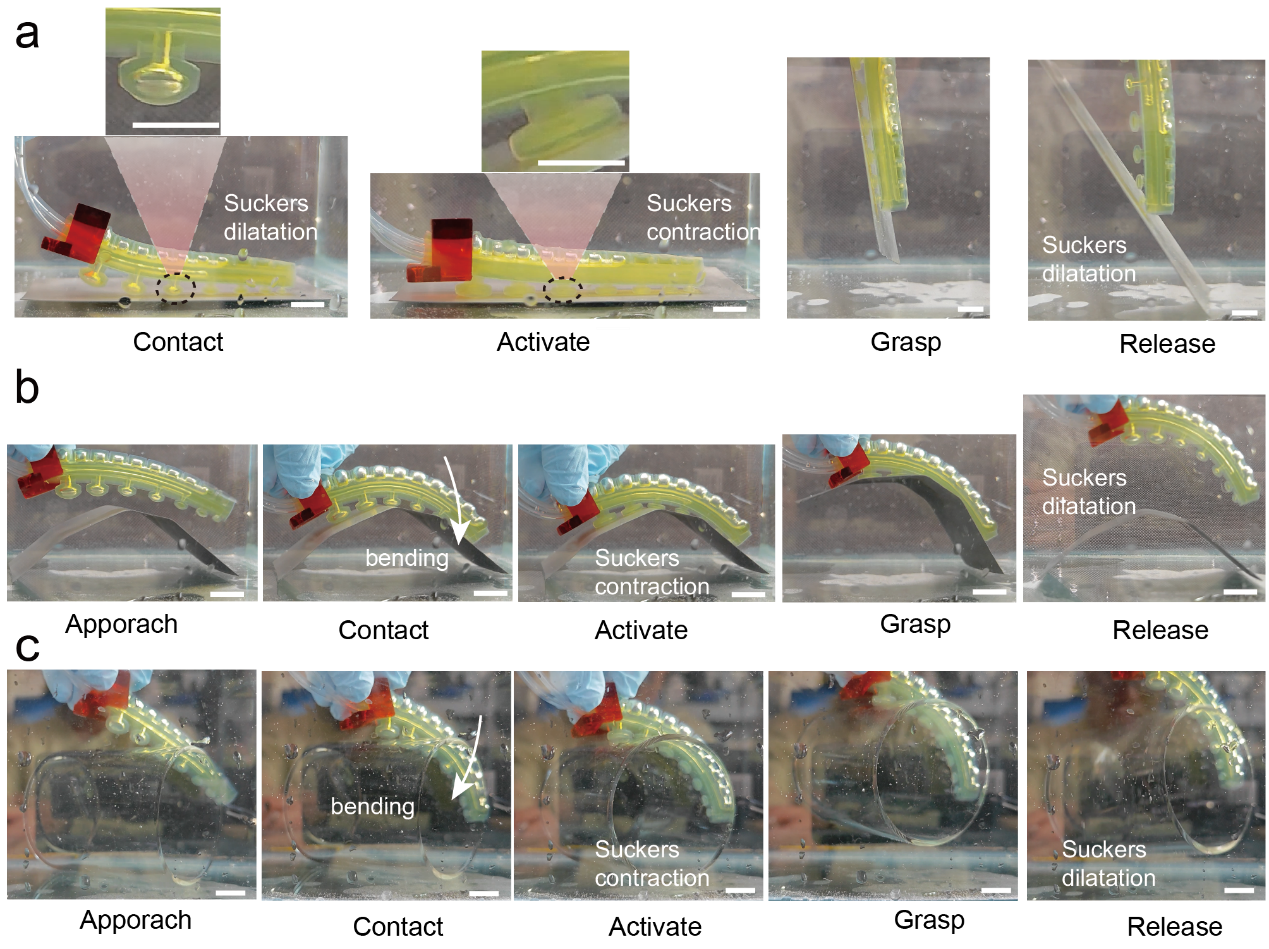


**Figure S17**. Photographs of the hydrogel gripper with suction cups manipulating objects.(a) Flat sheet. (b) Irregular sheet. (c) Glass jar. Scale bar: 10mm.


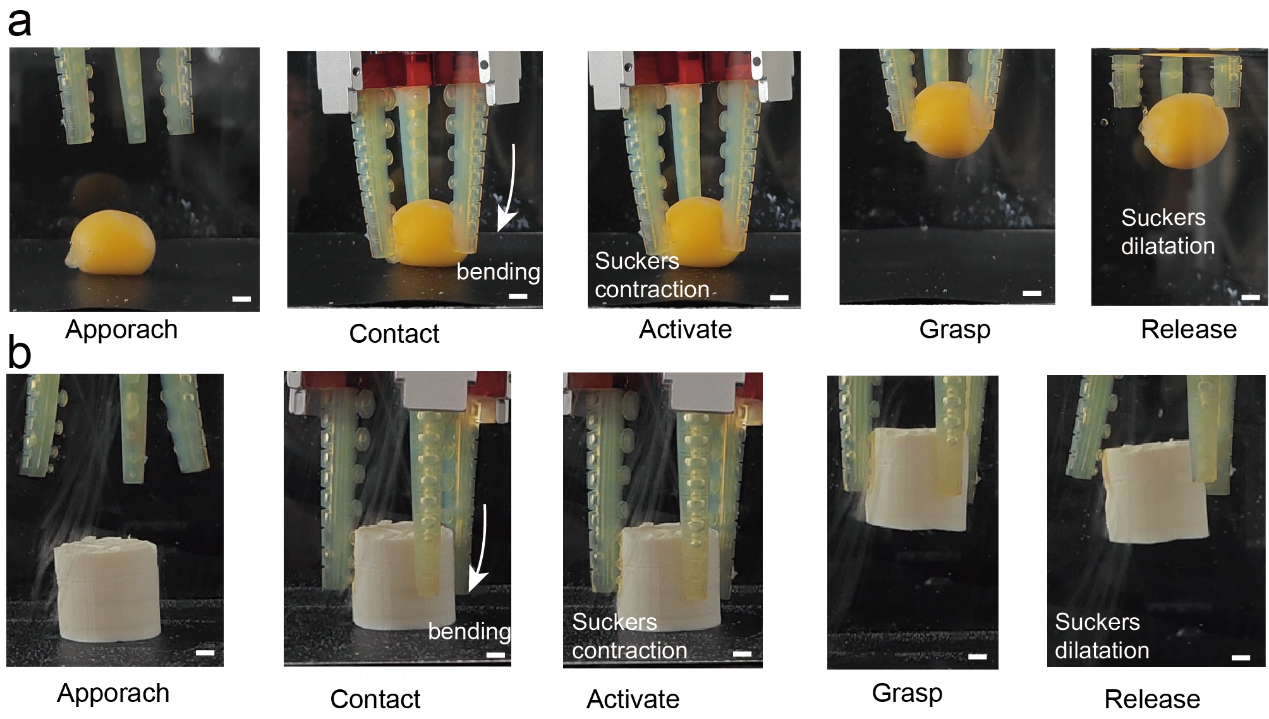


**Figure S18**. The process of grasping and releasing egg yolk and tofu with hydrogel grippers. (a) eff yolk.(b) tofu. Scale bar: 10mm.


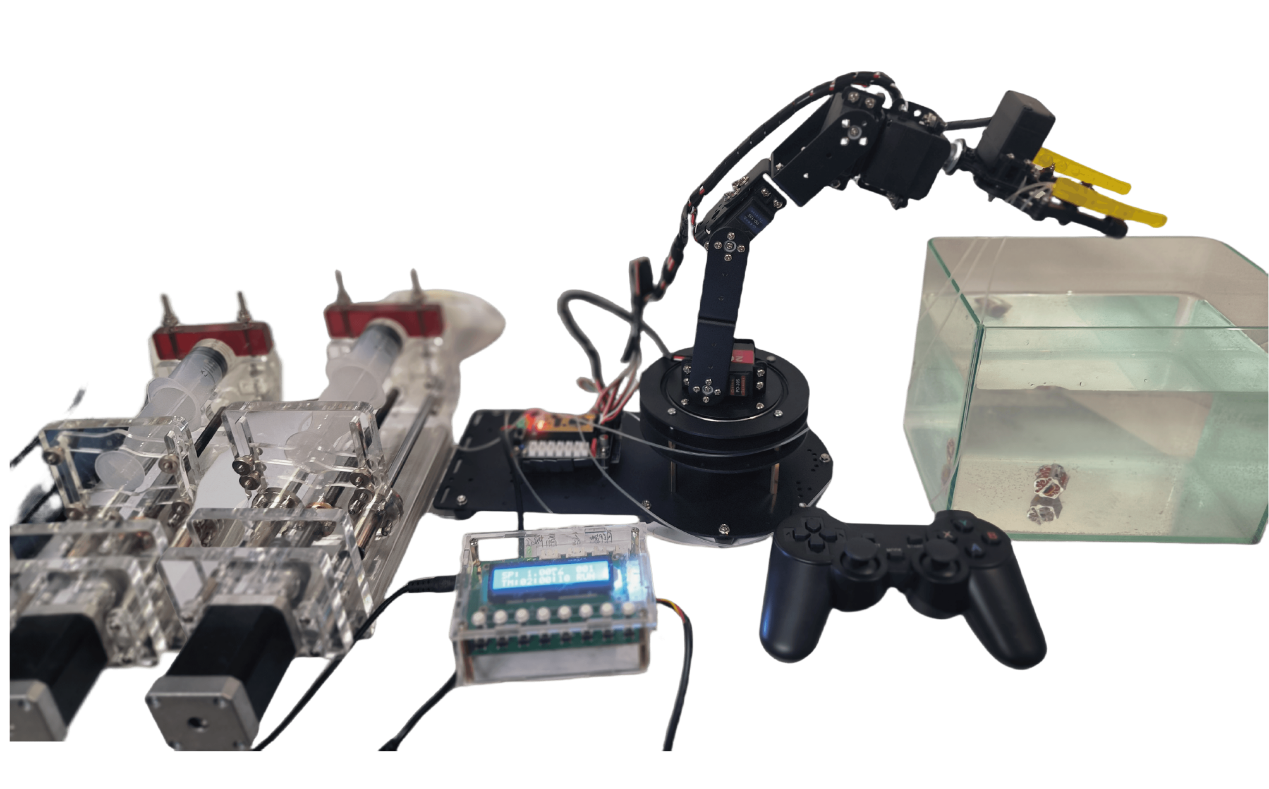


**Figure S19**. Physical photograph of the robotic arm hydrogel gripper system.


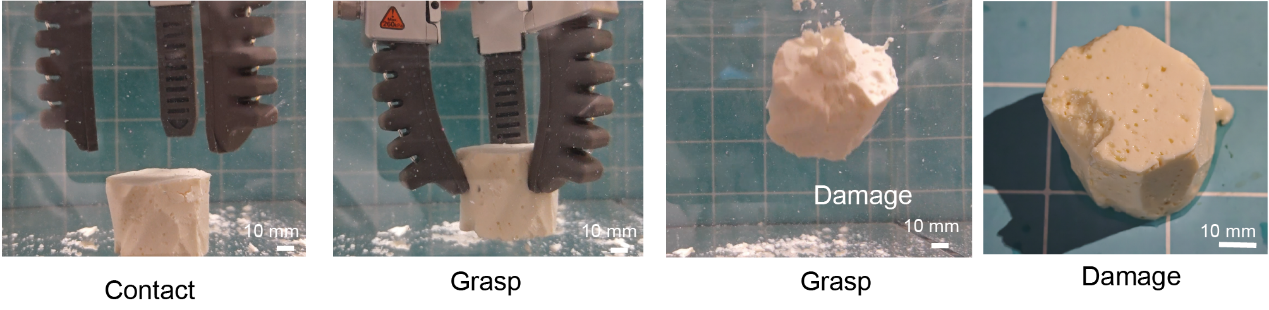


**Figure S20**. Photographs of the silicone gripper failing to grasp tofu.


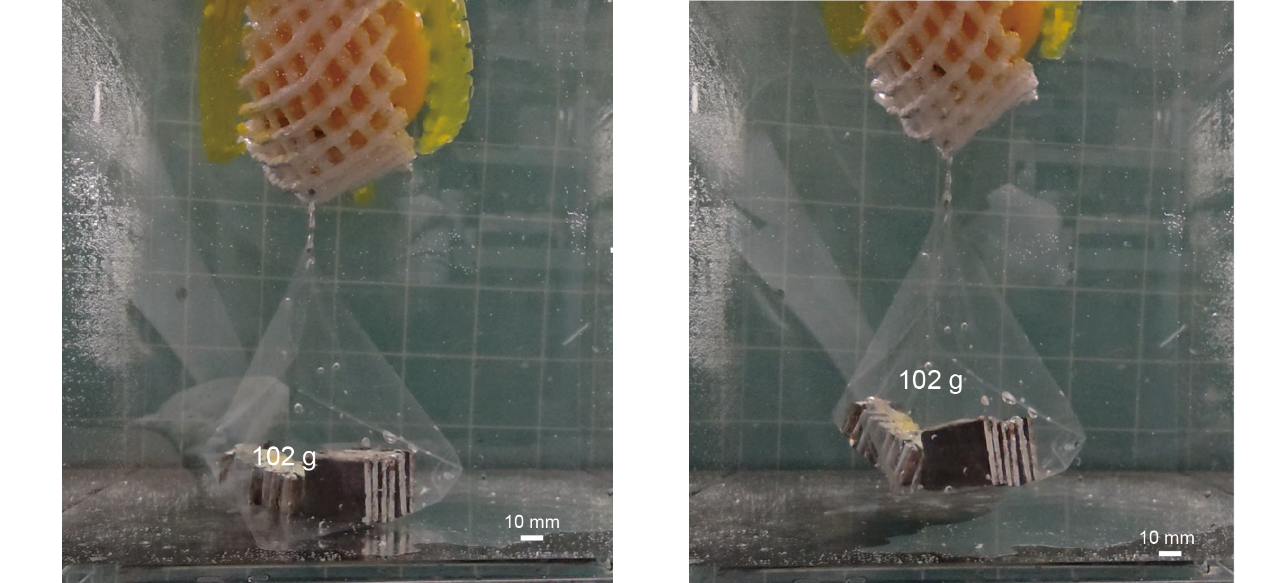


**Figure S21**. Photographs of the hydrogel gripper grasping a 102g weight.


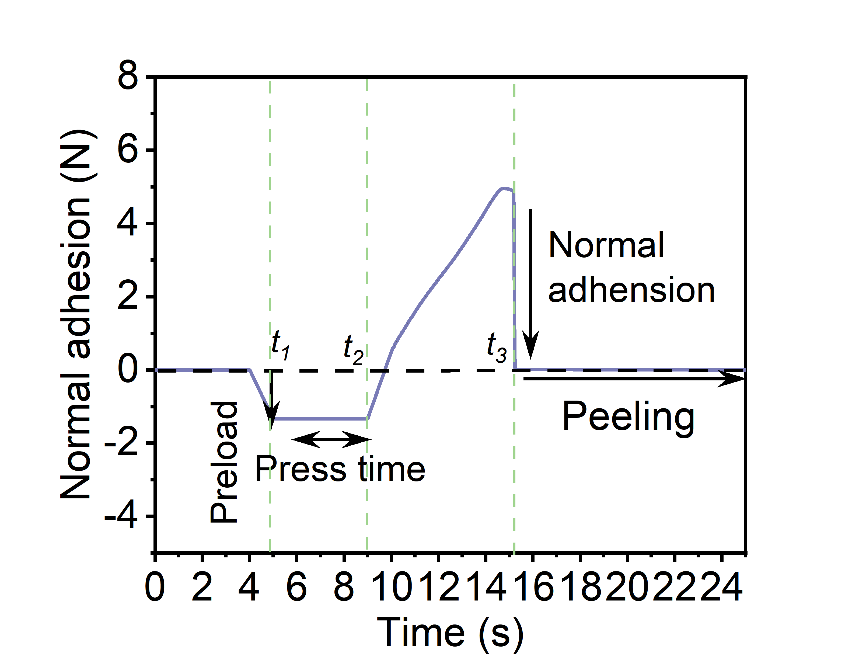


**Figure S22**. Normal adhesion test for hydrogel array suckers.
